# Integrating Machine Learning-Based Pose Sampling with Established Scoring Functions for Virtual Screening

**DOI:** 10.1021/acs.jcim.5c00380

**Published:** 2025-05-09

**Authors:** Thi Ngoc Lan Vu, Hosein Fooladi, Johannes Kirchmair

**Affiliations:** 1 Department of Pharmaceutical Sciences, Division of Pharmaceutical Chemistry, Faculty of Life Sciences, 27258 University of Vienna, Josef-Holaubek-Platz 2, 1090 Vienna, Austria; 2 Christian Doppler Laboratory for Molecular Informatics in the Biosciences, Department of Pharmaceutical Sciences, 27258 University of Vienna, Josef-Holaubek-Platz 2, 1090 Vienna, Austria; 3 Vienna Doctoral School of Pharmaceutical, Nutritional and Sport Sciences (PhaNuSpo), 27258 University of Vienna, Josef-Holaubek-Platz 2, 1090 Vienna, Austria

## Abstract

Physics-based docking methods have long been the cornerstone of structure-based virtual screening (VS). However, the emergence of machine learning (ML)-based docking approaches has opened new possibilities for enhancing VS technologies. In this study, we explore the integration of DiffDock-L, a leading ML-based pose sampling method, into VS workflows by combining it with the Vina, Gnina, and RTMScore scoring functions. We assess this integrated approach in terms of its VS effectiveness, pose sampling quality, and complementarity to traditional physics-based docking methods, such as AutoDock Vina. Our findings from the DUDE-Z benchmark dataset show that DiffDock-L performs competitively in both VS performance and pose sampling in cross-docking settings. In most cases, it generates physically plausible and biologically relevant poses, establishing itself as a viable alternative to physics-based docking algorithms. Additionally, we found that the choice of scoring function significantly influences VS success.

## Graphical abstract

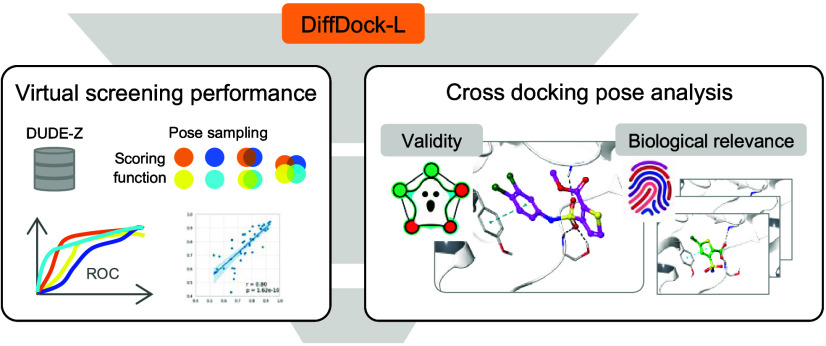

## Introduction

Physics-based docking methods have been at the forefront of structure-based protein–ligand interaction prediction and virtual screening (VS) for decades.^1^ Most of these approaches consist of two main components: an algorithm for pose sampling and a scoring function for pose evaluation.^1,2^ Physics-based docking approaches primarily rely on molecular mechanics force fields, but they also utilize information derived from protein structural data, mainly X-ray crystallographic data on protein–ligand complexes, and measured ligand binding affinities.^3^

Recent years have witnessed significant improvements in physics-based molecular docking through machine learning (ML) approaches. For example, ML approaches have been successfully employed as a rapid means for preselecting compounds for molecular docking, enabling the screening of huge molecular libraries with physics-based docking methods.^4,5^ ML-based scoring functions have shown favorable accuracy and computational efficiency in VS applications.^6,7^ Furthermore, ML-based pose sampling approaches have emerged as promising alternatives to traditional conformational sampling techniques, gaining considerable attention in the field.^1,8^

ML-based pose sampling approaches explore ligand poses using regression-based modeling (examples include EquiBind,^9^ TANKBind,^10^ E3Bind,^11^ KarmaDock,^12^ FlexPose,^13^ and CarsiDock^14^) or generative modeling (examples include DiffDock^15^ and its successor, DiffDock-L,^16^ as well as SurfDock^17^ and FlowDock^18^). Benchmark studies indicate that ML-based approaches can predict the orientation and conformation of protein-bound ligands.^9–12,15–18^ However, critique has been voiced about some conceptual limitations of benchmark studies that may result in overestimating pose prediction performance.^19–21^

Recent works have begun to explore ML-based pose sampling approaches for VS applications.^12,17,18,22,23^ For example, the developers of SurfDock successfully employed their method to identify novel small-molecule inhibitors of aldehyde dehydrogenase 1 family member B1 (ALDH1B1).^17^ Likewise, the developers of KarmaDock identified leukocyte tyrosine kinase (LTK) inhibitors with their docking approach.^12^ Most recently, a benchmark study on ML-based pose sampling approaches was reported, further highlighting the potential of these methods in VS applications.^23^

In this work, we thoroughly assess the VS performance and pose sampling performance of a VS approach integrating DiffDock-L^16^ with established scoring functions. We analyze the VS performance of various setups to explore the roles of the pose sampling and scoring elements, as well as the complementarity between ML-based and traditional pose sampling methods. Furthermore, we investigate—for the first time—the capability of the ML-based method in sampling relevant poses for cross-docking scenarios. Given concerns about the quality of the poses sampled by ML-based docking methods for redocking tasks,^19,23,24^ we study the validity and plausibility of the ML-based docking poses and compare them to those generated by physics-based docking methods.

DiffDock-L is one of the most popular deep-learning models for docking. Not only has it shown competitive performance among ML-based docking programs regarding pose prediction accuracy,^16^ physicochemical plausibility,^19^ and protein–ligand interaction recovery rate,^24^ but it is also one of the very few algorithms that support blind docking (i.e., docking without the necessity to define a ligand binding site).

DiffDock-L reports a confidence score for each generated docking pose that quantifies the likelihood of a pose having an RMSD of less than 2.0 Å to the hypothetical measured binding pose. This confidence score is ligand-specific and can only be used to rank poses of the same ligand. To enable VS, we supplemented DiffDock-L’s pose sampling and scoring capabilities with the Vina scoring function (from the AutoDock Vina package^25,26^ ), the Gnina scoring function,^27^ and RTMScore.^6^

We evaluate the VS performance of the approach integrating DiffDock-L on the DUDE-Z benchmark dataset^28^ and compare it to AutoDock Vina (from here on referred to as ‘Vina’) (see Figure 1). DUDE-Z is the third and latest generation of a well-established dataset for testing structure-based algorithms for their VS capacity. The benchmark dataset covers 43 structurally diverse and pharmaceutically relevant proteins, including kinases, proteases, G protein-coupled receptors (GPCRs), and nuclear receptors (see Methods for details). Vina is one of the most widely employed physics-based docking methods.

**Figure 1:**
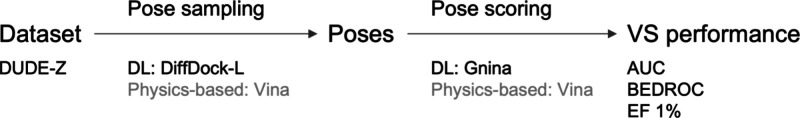
VS pipeline integrating DiffDock-L for pose sampling with the Vina and Gnina scoring functions for compound ranking. AutoDock Vina serves as a reference method for comparing the integrated approach with a physics-based docking approach.

## Methods

### Dataset

DUDE-Z served as the benchmark dataset, encompassing 43 pharmaceutically relevant protein targets. Each target is represented by 26 (FA10) to 123 (PARP1) experimentally verified ligands (Figure S1), with activities measured as EC_50_, IC_50_, *K*_i_, or *K*_d_ values of 1 μM or better. For each active compound, the dataset includes up to 50 carefully selected decoy molecules. These decoys are chosen based on property matching with the active compounds, considering physicochemical properties such as molecular weight, number of hydrogen bond donors and acceptors, number of rotatable bonds, and water–octanol partition coefficient (log P). As an improvement over previous versions of the benchmark dataset, the DUDE-Z decoy matching procedure ensures that the known active compound and its corresponding decoys possess identical net charges at physiological pH. Additionally, to guarantee that the decoys exhibit dissimilar topology from the active compounds, only molecules with an ECFP4-based Tanimoto similarity of up to 0.35 to any active compound are chosen as decoys. In total, DUDE-Z comprises 2,312 unique active ligands and 69,994 decoy compounds across the 43 target proteins.

The following data were collected from DUDE-Z for docking:

- 3D protein structural information deposited in the rec.crg.pdb files
- 3D structural information on the cocrystallized ligand deposited in the xtal-lig.pdb files
- 2D structural information on known ligands and decoys, deposited in ‘ligand.smi’ and ‘decoy.smi’, respectively, in the form of isomeric SMILES notations, with protonation states calculated for the physiologically relevant pH.

### Docking

#### Docking with DiffDock-L

For docking with DiffDock-L, fresh copies of the PDB files listed in the DUDE-Z were obtained directly from the PDB and loaded into the Schrödinger Platform (version 2021-1) for preprocessing using the Protein Preparation Wizard. The preparation steps included assigning bond orders, adding hydrogens, filling in missing side chains, removing water molecules, protonating residues at pH 7.4 using PROPKA, and restrainedly minimizing hydrogens using the OPLS4 ^29^ force field.

Docking with DiffDock-L was executed using the “inference.py” script (part of DiffDock-L) to generate 30 poses for each ligand. All other arguments were kept at default values specified in “default_inference_args.yaml” (part of DiffDock-L). Further details are provided in Table S1.

#### Docking with Vina

The representations of the proteins were prepared with the prepare_receptor4.py script (part of AutoDockTools^30^). This preparation step includes the addition of hydrogen atoms (*-A hydrogens*) and Gasteiger charges (default setting), as well as the removal of water molecules and chains composed entirely of residues other than the standard amino acids (default setting).

For each protein structure, the ligand binding site was defined as a box measuring 30 Å in each dimension, centered on the centroid of the cocrystallized ligand. The centroid was computed with the RDKit Chem.rdMolTransforms.​ComputeCentroid function, considering the heavy atom coordinates (note that all protein structures of DUDE-Z have exactly one cocrystallized ligand, regardless of the number of ligand binding pockets present in the structure).

For docking with Vina, the compound SMILES notations provided with DUDE-Z were transformed into RDKit^31^ mol objects and preprocessed with the same toolkit. Hydrogen atoms were added according to the protonation states provided by DUDE-Z. The molecules then went through sanitization (i.e., detecting chemistry errors and standardizing structural properties) and embedding (i.e., adding 3D coordinates), and an optimized 3D conformation was generated using the MMFF94 force field.^32^ Subsequently, atom and bond types were assigned with the MoleculePreparation.prepare function of the Meeko software package^33^ (to ensure compatibility with Vina). The prepared molecule structures were stored in .pdbqt format, ready for docking.

Docking with Vina was performed using Python scripts with default settings, except for the following: the exhaustiveness parameter, which indicates the number of conformation searches to run in parallel, was set to 32, and the number of poses generated for each compound was 30. A complete list of docking parameters used for docking with Vina is available in Table S1.

### Scoring of Ligand Poses

#### Scoring with the Vina Scoring Function

Poses obtained with the Vina docking algorithm were scored directly with the Vina scoring function. Poses generated with DiffDock-L were first subjected to local energy minimization (--local_only --minimize) and then scored with the Vina scoring function via the smina^34^ user interface.

#### Scoring with the Gnina Scoring Function

Poses generated with the Vina docking algorithm were scored directly with the Gnina scoring function (--cnn_scoring rescore). Poses generated with DiffDock-L were subjected to local energy minimization (--minimize) and then scored with the CNN scoring function of Gnina (--cnn_scoring rescore).

#### Scoring with the RTMScore

The docking poses sampled with Vina and the minimized poses generated with DiffDock-L were scored with the RTMScore using the “rtmscore.py” script provided in the RTMScore github repository under the “example” folder (https://github.com/sc8668/RTMScore/tree/main/example). Further details about scoring with RTMScore are provided in Supporting Information under “Exploring the RTMScore scoring function”.

### Evaluation of Virtual Screening Performance

The area under the ROC curve (AUC), BEDROC score, and enrichment factor among the 1% top-ranked compounds^35^ (EF1%) were calculated with the RDKit ML.Scoring.Scoring module. For BEDROC, the alpha value was set to 80.5 to have 2% of top-ranked compounds account for 80% of the score.

### Pose Analysis and Comparison

The docking poses obtained with Vina and the minimized docking poses obtained with DiffDock-L docking were checked for validity with PoseBusters. PoseBusters performs 18 checks to assess the chemical validity and consistency, as well as the intra- and intermolecular validity of docking poses.^19^ Eventually, the Boolean output PB_valid was checked, describing whether a pose passes all PoseBuster checks.

In addition, the protein–ligand interaction patterns of the docking poses were compared to those observed in a comprehensive set of measured structural data of protein–ligand complexes of the same target protein (“reference set”) using the protein–ligand interaction fingerprint (PLIF). The reference set of experimentally determined protein–ligand structures was obtained from the Protein Data Bank (https://www.rcsb.org/) with SIENA^36^ using the “ligand pose comparison” mode. In ligand pose comparison mode, SIENA retrieves all measured protein–ligand complexes with backbone conformations similar to the query structures (i.e., the structures provided by DUDE-Z). The structures retrieved with SIENA were filtered for binding sites with a “binding site identity” of 1.0 (meaning that the amino acids forming the binding site of the retrieved structure are identical to those forming the binding site of the query structure). The remaining protein–ligand structures were parsed with the MDAnalysis package,^37,38^ and the ligand structures were extracted. Both the protein and ligand structures were protonated at pH 7.4 using OpenBabel.^39^ Subsequently, the ligands were locally minimized inside the respective binding sites with the Vina scoring function via the smina^34^ interface.

In preparation for PLIF generation, the amino acids of the reference structures were adjusted to match those of the query structures. This step ensures the bit identity of the generated PLIFs, enabling direct comparison and similarity assessment.

PLIFs were generated from all protein–ligand complexes predicted with docking or retrieved by SIENA using the ProLIF package.^40^ As required by ProLIF, explicit hydrogens were added to the proteins and ligands using OpenBabel before calculating PLIFs. The following interaction types were considered: hydrogen bonds (“HBAcceptor” and “HBDonor”), ionic (“Anionic” and “Cationic”), metal coordination (“MetalAcceptor” and “MetalDonor”), cation−π (“CationPi” and “PiCation”), π–π stacking (“PiStacking”), hydrophobic (“Hydrophobic”) and halogen bond (“XBAcceptor” and “XBDonor”). The other three interaction types supported by the ProLIF package were excluded as they are either not sufficiently descriptive (van der Waals radii contacts) or too specific (edge-to-face and face-to-face π–π stacking) for this study.

## Results

This work integrates various pose sampling methods and scoring functions for VS applications. For concise reporting, we denote the explored setups as ‘[pose sampling]+​[scoring]’. For instance, ‘DiffDock-L+Gnina’ describes the VS in which poses are sampled using DiffDock-L and scored with the Gnina scoring function.

### Comparative Assessment of Virtual Screening Performance

#### Overall and Target-Specific Virtual Screening Performance

Using AUC to measure VS success across the 43 DUDE-Z targets, DiffDock-L achieved average values of 0.76 with the Vina scoring function and 0.82 with the Gnina scoring function (Figure 2). In comparison, when Vina was used as the pose sampling method, the average AUCs obtained with the Vina and Gnina scoring functions were 0.75 and 0.84, respectively. These results suggest that the VS performance using DiffDock-L as the pose sampling method is comparable to the Vina approach.

**Figure 2:**
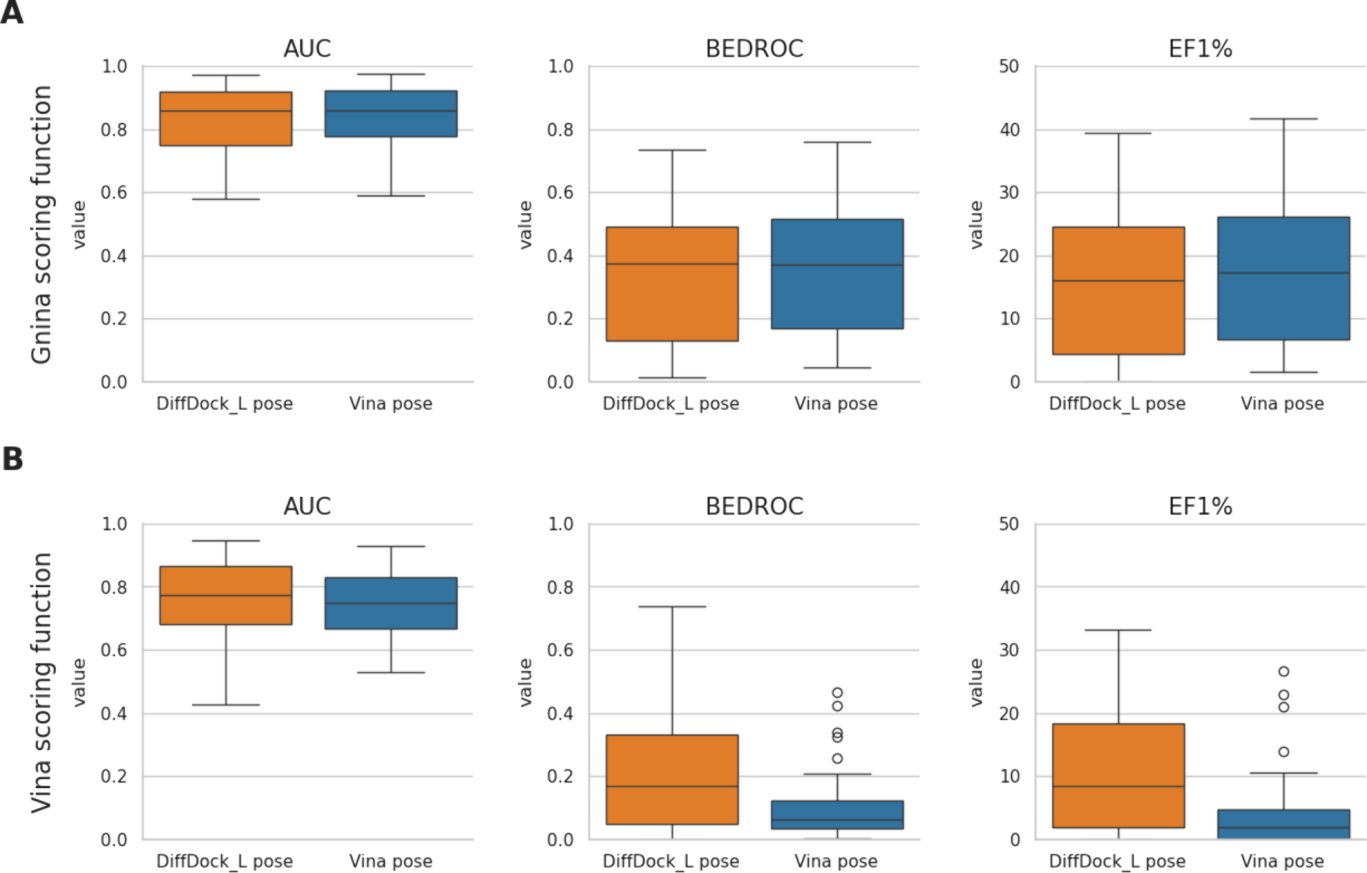
VS performance on the DUDE-Z dataset using the pose sampling method DiffDock-L (orange boxes) in combination with (A) the Gnina scoring function and (B) the Vina scoring function. The respective results with Vina pose sampling (blue boxes) are shown for comparison.

For all four combinations of pose sampling and scoring functions analyzed, the performance of VS varied across the 43 DUDE-Z targets. This variability is illustrated by the observed AUCs (Figure 2 and Table 1), BEDROC scores (Figure 3 and Table 1), and EF1% values (Table 1). While the AUC serves as a good indicator of the overall discriminative power of a VS approach, BEDROC and EF1% concentrate on quantifying early enrichment, meaning the method’s ability to rank active compounds highly in the hit list (e.g., within the top 1% of ranks).

**Figure 3:**
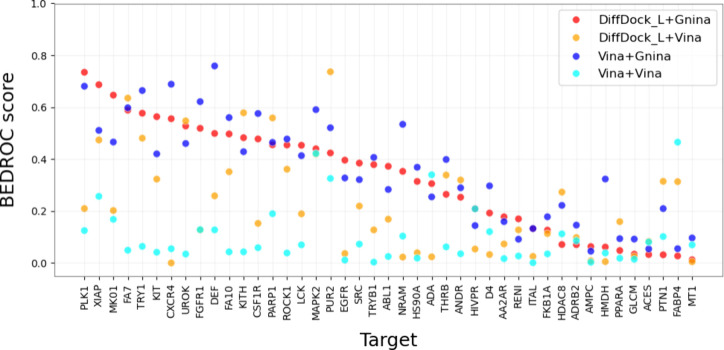
BEDROC scores achieved by individual VS setups for each target, sorted by decreasing performance of the DiffDock-L+Gnina combination.

**Table 1:** VS Performance Obtained with Different Combinations of Pose Sampling and Scoring Methods^a^

**VS setup**	**AUC**	**BEDROC** ^b^	**EF1%**	**no. targets with** BEDROC score ≥0.5	**% known active scaffolds recovered** among the top 1% ranks
DiffDock-L+Gnina	0.82 ± 0.10	0.33 ± 0.21	16.22 ± 11.86	9	17 ± 12
DiffDock-L+Vina	0.76 ± 0.13	0.22 ± 0.20	10.92 ± 10.50	5	11 ± 10
Vina+Gnina	**0.84 ± 0.10**	**0.36 ± 0.20**	**17.88 ± 11.93**	**12**	**19 ± 13**
Vina+Vina	0.75 ± 0.11	0.10 ± 0.11	3.89 ± 6.30	0	4 ± 6

a Best results indicated in bold.

b BEDROC score calculated with *a* = 80.5.

Considering BEDROC scores of ≥0.5 indicative of excellent VS performance, the combination of DiffDock-L and Gina yielded good screening results for 9 out of 43 targets. In comparison, pose sampling with Vina achieved excellent results for 12 targets. When Vina served as the scoring function, DiffDock-L obtained excellent results for 5 targets, while Vina achieved excellent results for none.

Among the top 1% ranks, DiffDock-L+Gnina recovered 17% of the active scaffolds. The recovery was higher for Vina+Gnina (19%) but lower for DiffDock-L+Vina (11%) and Vina+Vina (4%), indicating a major impact of the scoring functions on VS performance.

When using the same scoring function, a strong correlation was observed between the AUC values obtained with the two pose sampling methods across the 43 protein targets (Figures 4A and 4B). With Gnina as the scoring function, the Pearson r between the AUC values for the two pose sampling methods was 0.75 (*p*-value < 10^–4^). With Vina as the scoring function, the Pearson correlation coefficient was 0.80 (*p*-value < 10^–4^).

**Figure 4:**
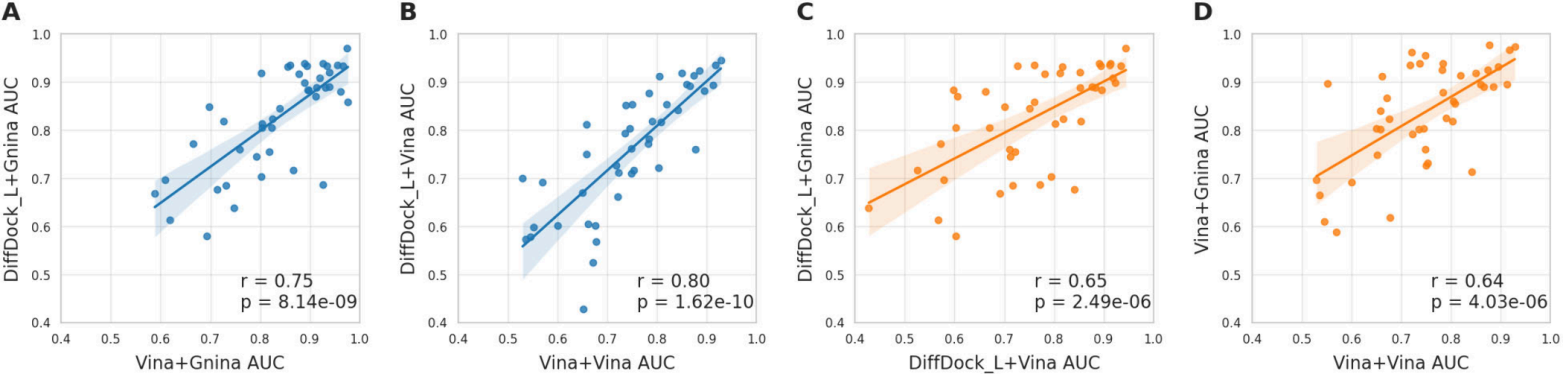
Correlations of the AUC values obtained for the 43 DUDE-Z targets using (A, B) the two pose generators with the same scoring function, and (C, D) the two scoring functions with the same pose generator. Stronger correlations are observed between docking setups using different pose generators but the same scoring function.

Weaker correlations were observed between the AUC values obtained with the same pose sampling method in conjunction with the two scoring functions (Figures 4C and 4D). Specifically, when using DiffDock-L as the pose sampling method, the AUC values from the two scoring functions produced a Pearson correlation coefficient of 0.65. With Vina poses, the correlation coefficient between the AUC values was 0.64.

Similar to the findings with the AUC, when paired with the Gnina scoring function, DiffDock-L achieved early enrichment performance comparable to Vina pose sampling, with average BEDROC scores of 0.33 and 0.36 and EF1% values of 16.22 and 17.88, respectively. However, when utilizing the Vina scoring function, DiffDock-L demonstrated superior performance compared to Vina pose sampling, achieving a BEDROC score of 0.22 versus 0.10 and an EF1% of 10.92 versus 3.89.

Strong correlations were observed for BEDROC and EF1% between the DiffDock-L and Vina pose sampling methods but only when combined with the Gnina scoring function (BEDROC Pearson r = 0.88, *p*-value < 10^–4^; EF1% Pearson r = 0.86, *p*-value < 10^–4^). In contrast, when combined with the Vina scoring function, the correlation between the VS results obtained from the two pose sampling methods was much weaker (BEDROC Pearson r = 0.30, *p*-value = 0.05; EF1% Pearson r = 0.06, *p*-value = 0.71).

#### Behavior and Impact of Scoring Functions on Virtual Screening Success

To understand the distinct behavior of the two scoring functions regarding BEDROC and EF1%, we investigated their performance on a per-molecule basis. We found a strong correlation between the Gnina scores derived from DiffDock-L and Vina poses (Pearson r and *p*-values are reported as part of Figure S2). However, we also observed that many compounds, particularly decoys, received substantially higher scores from the Vina scoring function for Vina poses than for DiffDock-L poses (see Figure 5 for examples and Figure S3 for a complete set of figures). While pinpointing the exact reason for this behavior is challenging, the VS process appears to benefit from independent pose generation and scoring processes (the Vina pose sampling algorithm employs the Vina scoring function to guide the sampling process toward the scoring function minima).

**Figure 5:**
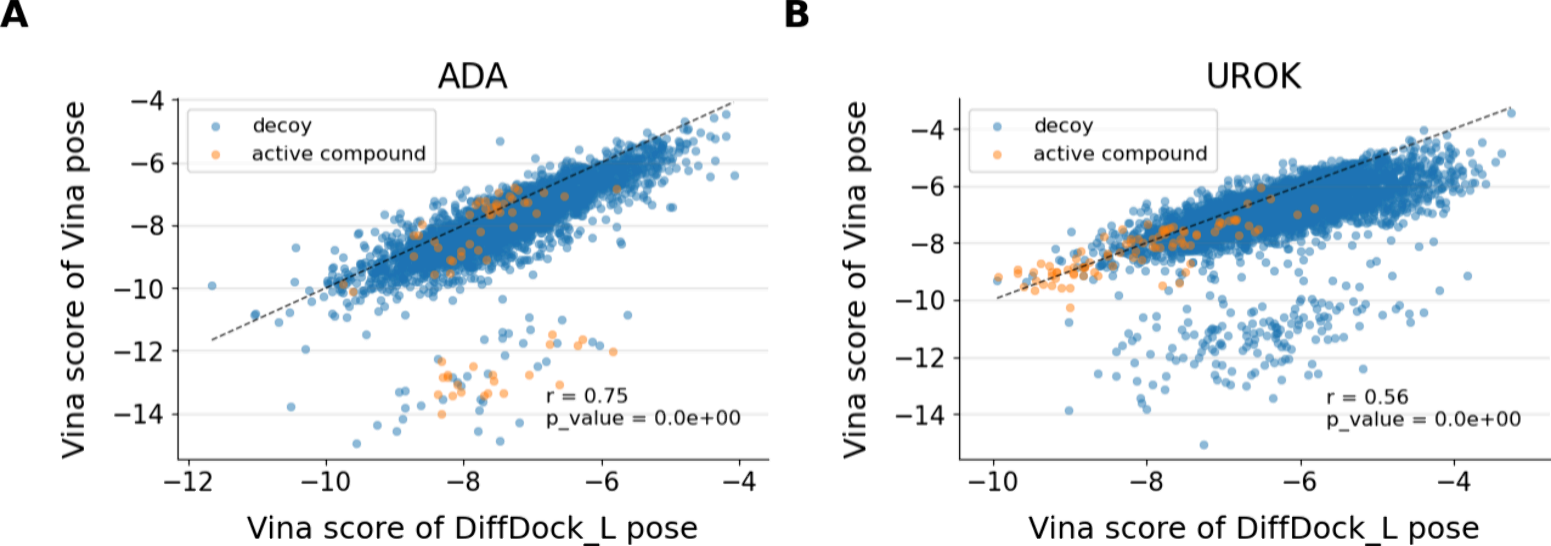
Vina scores assigned to individual molecules for two representative targets included in DUDE-Z: (A) ADA and (B) UROK. ADA is the target where the most significant improvement in EF1% was observed by switching pose sampling from DiffDock-L to Vina. Many compounds active on ADA were correctly assigned as favorable, i.e., negative Vina scores for the poses generated by Vina (orange data points located in the lower center of plot (A)). In contrast, UROK is the target where the largest improvement in EF1% was recorded by switching pose sampling from Vina to DiffDock-L. Many decoys for UROK were incorrectly assigned favorable Vina scores for the poses generated by Vina (blue data points located in the lower center section of plot (B)).

Due to the observed significance of the scoring function in VS performance, we investigated the behavior of a third popular scoring function, the RTMScore. RTMScore utilizes a residue-based graph representation strategy, employing multiple graph transformer layers for representation learning and a mixture density network to derive the residue-atom distance likelihood potential. RTMScore has demonstrated strong performance across various VS contexts applications.^6,23^

As shown in Figure S4, the VS performance of the pose sampling methods combined with the RTMScore was comparable to VS setups employing the Vina and Gnina scoring functions. Strong correlations were observed between the VS performance of the two pose sampling methods combined with the RTMScore (Peason r = 0.77, 0.85, and 0.83 for AUC, BEDROC, and EF1%, respectively; see Figure S5). These correlations are consistent with the ones observed when using Gnina as the scoring function. Overall, these observations further underscore the substantial influence of scoring functions on VS performance.

#### Virtual Screening Performance in Different Chemical and Protein Spaces

To gain insights on the performance of the VS setups in different molecular contexts, we dissected the methods’ behavior from a small-molecule and a target protein perspective.

For the compound-focused analysis, we divided the compound sets based on three key physicochemical properties (Figure S6): molecular weight (MW), hydrophobicity (calculated as log P), and the number of rotatable bonds (RBs). For each of these properties, we generated three subsets representing the lower, middle, and upper ranges. By comparing the methods’ performance on the lower and upper thirds of the datasets to the full dataset, we assessed each method’s capabilities across distinct chemical spaces. To ensure validity, we excluded targets with fewer than 10 active compounds in either the lower or upper subset from this analysis. Applying this criterion resulted in the consideration of 16, 25, and 22 targets for the analysis related to MW, log P, and RBs, respectively.

Across the selected targets, DiffDock-L and Vina exhibited similar behavioral patterns regarding the three physicochemical properties (Figures 6 and S7). VS performance decreased with increasing MW for all combinations of pose sampling and scoring methods. This performance decline can be attributed to the correlation between MW and molecular/geometric complexity and, as a consequence, the hardness of the docking problem.

**Figure 6:**
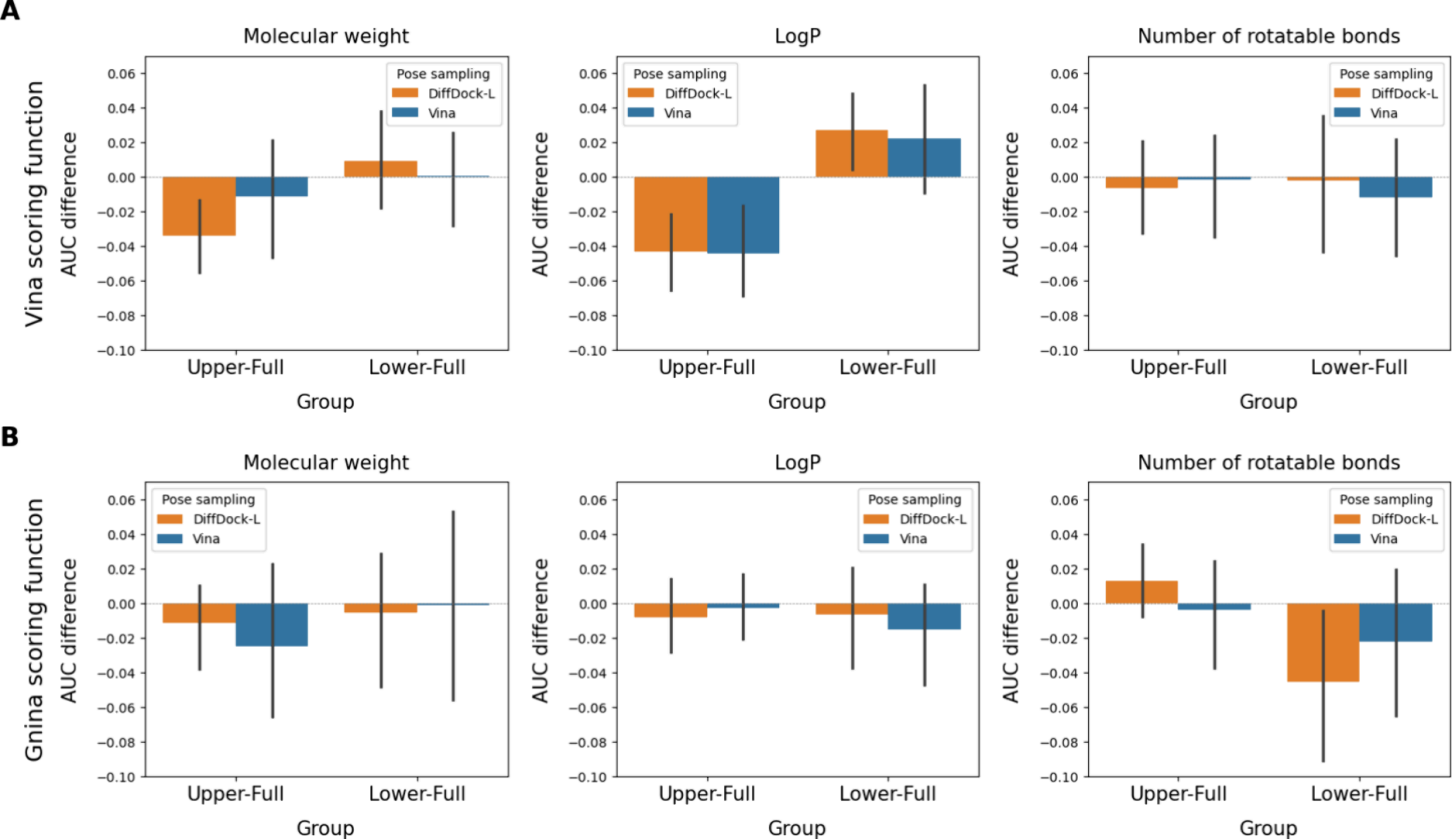
Difference in VS performance (measured as ΔAUC) between the compound subsets representing the lower/upper thirds of compounds (with respect to one of three key physicochemical properties) and the full datasets. The analysis related to MW, log P, and RBs was performed with 16, 25, and 22 targets, respectively.

Both pose sampling methods demonstrated sensitivity to molecular hydrophobicity, particularly with the Vina scoring function. Compared to the full dataset, performance was lower for hydrophobic compounds. This trend aligns with expectations since hydrophobic interactions lack directionality compared to interactions such as hydrogen bonds, which offer better-defined geometric constraints that aid binding pose identification by docking algorithms.

In conjunction with the Vina scoring function, the VS performance of the two pose sampling methods did not significantly differ for compounds with many or few RBs. However, both DiffDock-L and Vina pose sampling exhibited decreased performance on compounds with fewer RBs when used with the Gnina scoring function.

Contrary to MW and log P, the RB-related performance was disproportionately affected by two outlier targets, GLCM and TRY1 (Figure S7). Upon investigating the datasets of these two targets, we discovered an accumulation of low MW ligands among the compounds with few RBs (in the lower third), compared to their corresponding decoys (Figure S8). This disparity in MW likely undermines proper molecular ranking, as higher MW compounds generally form more interactions with target proteins, resulting in better scoring outcomes. Consequently, the performance pattern related to the number of RBs may reflect MW differences rather than direct RB effects on docking accuracy.

To understand the observed variations in VS performance from a target protein perspective, we analyzed key properties of the ligand binding sites that often determine their docking complexity, such as pocket depth, hydrophobicity, and solvent exposure (see Supporting Information subsection “Binding site properties analysis” and Table S2 for a comprehensive overview). However, no consistent patterns emerged (Figure S9), which impeded definitive conclusions about the relationship between binding site properties and method performance.

Furthermore, we investigated whether DiffDock-L’s performance might be influenced by prior exposure to related protein structures. DUDE-Z targets share Evolutionary Classification of protein Domains (ECOD) annotations with proteins in DiffDock-L’s training data, indicating high structural similarity. The availability of this information prompted us to curate a new test set with ECOD annotations absent from DiffDock-L’s training data and a sufficient number of active compounds for VS performance evaluation.

Despite extensive efforts, we were left with only three suitable candidate protein targets that satisfy the two quality criteria we applied (i.e., protein structure distinct from any structure in the DiffDock-L training set; ≥32 active compounds), highlighting the scarcity of appropriate test cases in the field (full details of the data curation process and the identified targets are available in the Supporting Information subsection “Protein similarity to DiffDock-L training data”). VS on these three targets revealed only marginal performance differences between the four combinations of pose sampling and scoring methods (Figure S11). The limited data available for benchmarking prevents us from drawing robust conclusions about method preferences across different target types.

### Hedging Strategy for Increased Success Rates in Virtual Screening

To enhance the early enrichment and robustness of structure-based VS, we investigated various consensus strategies that determine the ranks of compounds based on predictions obtained using DiffDock-L and Vina pose sampling methods in combination with either or both scoring functions.

Among all the consensus strategies explored (as reported in Table 2), averaging the ranks of compounds obtained with DiffDock-L+Gnina and Vina+Gnina performed the best. With an average BEDROC score of 0.38 and an average EF1% of 19.33 across the 43 targets, this consensus approach surpassed the VS setups using either DiffDock-L or Vina individually. The consensus scoring method achieved, on average, the highest ratio of active scaffolds (21%) among the top 1% of ranks. Additionally, it increased the number of targets for which BEDROC scores of ≥0.5 were reported from 12 (the highest number achieved by the individual VS setups) to 17.

**Table 2:** VS Performance Obtained with Different Consensus Approaches^a^

	DiffDock-L pose sampling		**Vina pose sampling**						
**Best/Average value** ^b^	**Gnina scoring**	**Vina scoring**	**Gnina scoring**	**Vina scoring**	**AUC**	**BEDROC** ^c^	**EF1%**	**no. targets with BEDROC score ≥0.5**	**% known active scaffolds recovered among the top 1% ranks**
Best	X		X		**0.85** ± 0.10	0.35 ± 0.20	17.49 ± 11.60	11	19 ± 13
		X		X	0.77 ± 0.11	0.18 ± 0.15	8.12 ± 7.77	2	8 ± 8
	X	X	X	X	0.84 ± 0.10	0.28 ± 0.16	12.98 ± 8.76	4	15 ± 10

Average	X		X		0.84 ± 0.10	**0.38** ± 0.22	**19.33** ± 13.24	**17**	**21 ± 14**
		X		X	0.77 ± 0.12	0.22 ± 0.19	10.33 ± 9.75	5	11 ± 10
	X	X	X	X	0.83 ± 0.10	0.35 ± 0.23	17.22 ± 12.45	14	18 ± 12

a Best results indicated in bold.

b Best or average value obtained with any of the methods indicated by X’s.

c BEDROC score calculated with *a* = 80.5.

Overall, these results suggest that VS benefits only marginally from the combination of DiffDock-L with Vina pose sampling integrated with the two scoring functions. One possible explanation is the strong correlation between the methods’ VS performance across individual targets (see the previous section).

### Pose Analysis

Now that we have established that the VS performance of DiffDock-L is comparable to Vina’s, we were interested in comparing the physical validity and plausibility of the ligand poses generated by the two pose sampling methods.

This analysis is driven by two key factors. First, VS performance metrics not only reflect the effectiveness of pose sampling methods but also show the influence of scoring functions, which significantly impact results, as indicated in earlier sections. Relying solely on VS performance metrics offers an incomplete view of pose sampling methods, emphasizing the need for a focused examination of their contributions.

Second, most current reports on the pose sampling capacity of ML-based approaches are confined to redocking exercises. In redocking, a ligand is inserted into its cognate protein structure, which represents a scenario of limited relevance to real-world applications. It is important to note that VS typically involves docking and recognizing structurally distinct compounds.

In the following experiments, we isolate the evaluation of pose sampling from scoring by assessing the ability of the pose sampling methods to generate valid and plausible poses for known active compounds in VS.

#### Physical Validity of Docking Poses

According to PoseBusters, DiffDock-L produced at least one physically valid pose for 95.53% of the active compounds included in DUDE-Z (Table 3 and Figure S12). Vina produced at least one physically valid pose for 99.50% of all active compounds. Given these high success rates, both pose sampling methods typically generate physically valid poses.

**Table 3:** Physical plausibility and similarity to the measured protein-ligand complexes of the pose sampling methods.

	**Pose sampling with**	
	DiffDock-L	**Vina**
Total number of docked active compounds from DUDE-Z	2,785 (100.00%)	2,790 (100.00%)
Number of compounds with at least one physically valid docking pose^a^	2,772 (99.53%)	2,776 (99.50%)
AND PLIF_sim^b^ ≥ 0.50	2,418 (86.82%)	2,670 (95.70%)
AND PLIF_sim^b^ ≥ 0.85	610 (21.90%)	830 (29.75%)
AND PLIF_sim^b^ = 1.00	139 (4.99%)	203 (7.28%)

a Physical validity according to PoseBusters.

b Maximum similarity (Tanimoto coefficient) of the PLIF derived from the docking pose and the PLIFs derived from any of the measured protein–ligand complexes of the corresponding target.

#### Plausibility of Protein–Ligand Interaction Patterns

In addition to physical validity, we aimed to understand how closely the generated docking poses resemble the protein–ligand interaction patterns observed in measured structural data. To achieve this, we compiled an extensive set of 2,501 protein–ligand complexes for the 43 targets of interest from the PDB (see Methods for details and Figure S13 for the number of protein–ligand complexes collected for each target). We subsequently generated the PLIFs for all these measured protein–ligand complexes and those created through docking (see Methods for details). This process allowed us to compare the PLIFs derived from both measured and generated protein–ligand complexes.

According to the generated PLIFs, the median number of protein–ligand interactions observed among the 2,501 measured protein–ligand complexes was 11, with substantial variation across the ligands and targets (Figure S14). In comparison, the median number of protein–ligand interactions identified among the docked poses of the active compounds of DUDE-Z was slightly lower (7 for DiffDock-L and 8 for Vina; Figure S15). This is plausible because detecting some of the protein–ligand interactions requires considering the conformational changes induced upon ligand binding to the target protein.

We then examined whether the generated docking poses resemble the protein–ligand interaction patterns of the measured protein–ligand complexes. To obtain informative results from this experiment, we prefiltered the 2,501 measured protein–ligand complexes to include only those forming at least four interactions based on their PLIFs. This filtering step reduced the number of measured protein–ligand complexes to 2,425.

On average, the PLIFs generated from DiffDock-L poses were less similar to those derived from the measured protein–ligand complexes than the PLIFs generated from Vina poses (Table 3). DiffDock-L produced at least one pose with high PLIF similarity to one or more measured protein–ligand complexes for 22% of the active compounds (of each target), while for Vina, this value was 30%. A PLIF similarity of 1.00 was observed with DiffDock-L and Vina for 5% and 7% of the active compounds, respectively.

Of course, the structural relatedness between the active compounds in DUDE-Z and the ligands represented by the measured structural data varies greatly and defines the maximum PLIF similarity that can be reached in this experiment. Considering these dependencies and limitations, the PLIF similarities obtained in our experiment are within expectations. We did not observe substantial differences in the ability of the two methods to generate poses resembling the measured structural data.

#### Similarity of Protein–Ligand Interaction Patterns and Ligand Structures

It is well-known that obtaining a correct docking pose for the cocrystallized ligand (redocking) is usually less challenging than for structurally distinct compounds. This is because docking algorithms have limitations in considering protein flexibility (specifically, the induced fit effect) and solvent dynamics. However, the true value of docking algorithms lies in their ability to identify new chemistry.

Here, we explore the similarity between measured and predicted protein–ligand interaction patterns (represented as PLIFs) as a function of molecular relatedness (quantified as the Tanimoto coefficient based on Morgan2 fingerprints with 2,048 bits). We expect the protein–ligand interaction patterns derived from measured data and docking poses to be more similar for structurally related compounds than those of structurally distinct ones. It is reasonable to assume that structurally related ligands generally form similar interactions with the same target protein.

Our data supports the assumption of a direct correlation between molecular similarity and PLIF similarity. For DiffDock-L poses of molecules that are structurally distinct from any measured ligand structures (with Tanimoto coefficients not exceeding 0.30), the median PLIF similarity was 0.55 (Figure 7; refer to Figure S16 for results on the individual targets). In contrast, for structurally related pairs of molecules (with Tanimoto coefficients greater than 0.75), the median PLIF similarity was 0.67, which is significantly higher (Welch’s *t* test *p*-value < 10^–4^). The results for DiffDock-L aligned with the observations for Vina. With a median of 0.67, the PLIF similarity was significantly higher (Welch’s *t* test *p*-value < 10^–4^) for the bin of structurally related pairs of compounds than for the bin of structurally distinct compounds (median 0.57).

**Figure 7:**
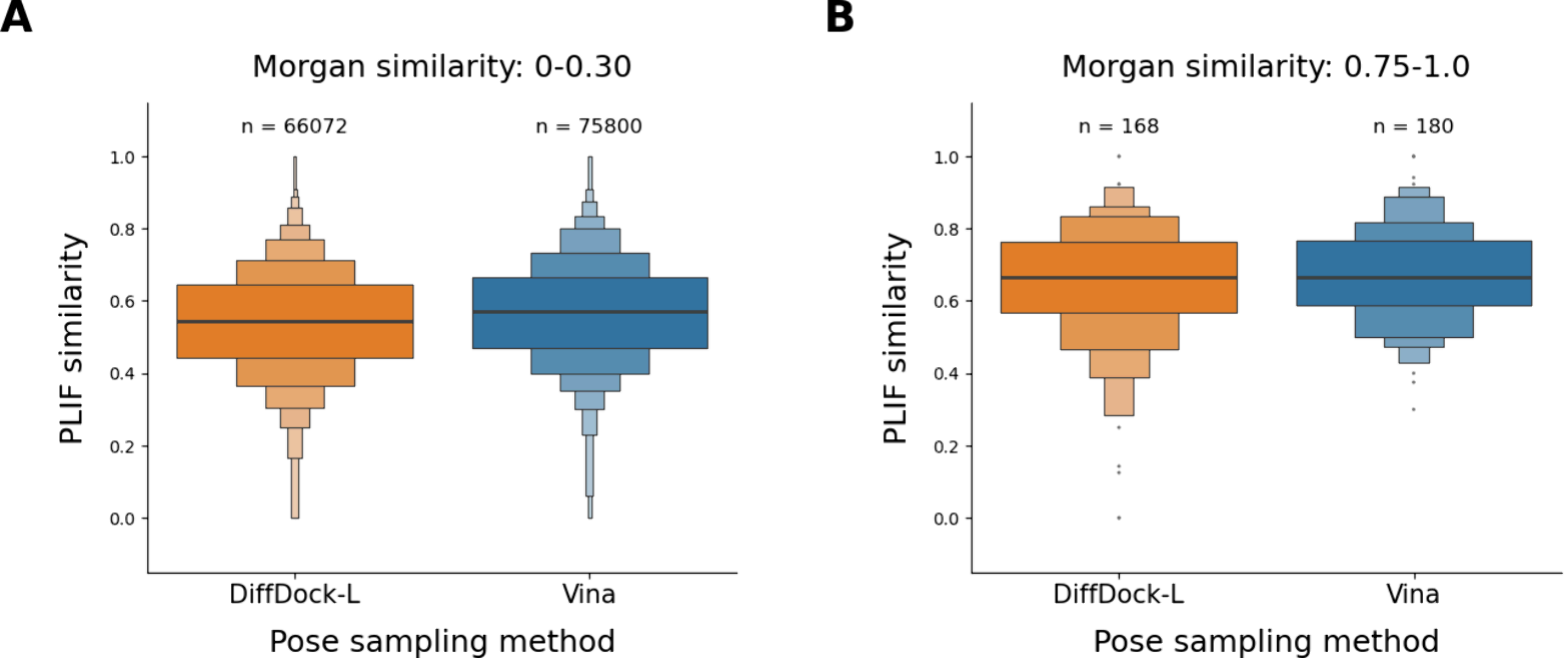
PLIF similarities between pairs of (A) structurally distinct measured and predicted protein–ligand complexes and (B) structurally related measured and predicted protein–ligand complexes sampled with DiffDock-L and Vina for 43 DUDE-Z targets.

In both the high and low-similarity bins, the differences in the averaged PLIF similarities of poses generated by DiffDock-L and Vina were minimal. Specifically, Welch’s *t* test *p*-value was smaller than 10^–4^, and Cohen’s d was 0.20 for the low-similarity bin. For the high-similarity bin, the Welch’s *t* test *p*-value was 0.10, and Cohen’s d was 0.18. These results indicate that the performance of the two pose sampling methods, when stratified by the structural relatedness of the docked compounds, is comparable.

During our investigations, we identified several cases of high PLIF similarity obtained for ligands that are structurally distinct from any cocrystallized ligands. These examples demonstrate the ability of docking methods to retrieve plausible binding poses for new chemistry. For DiffDock-L, two such examples are illustrated in Figure 8.

**Figure 8:**
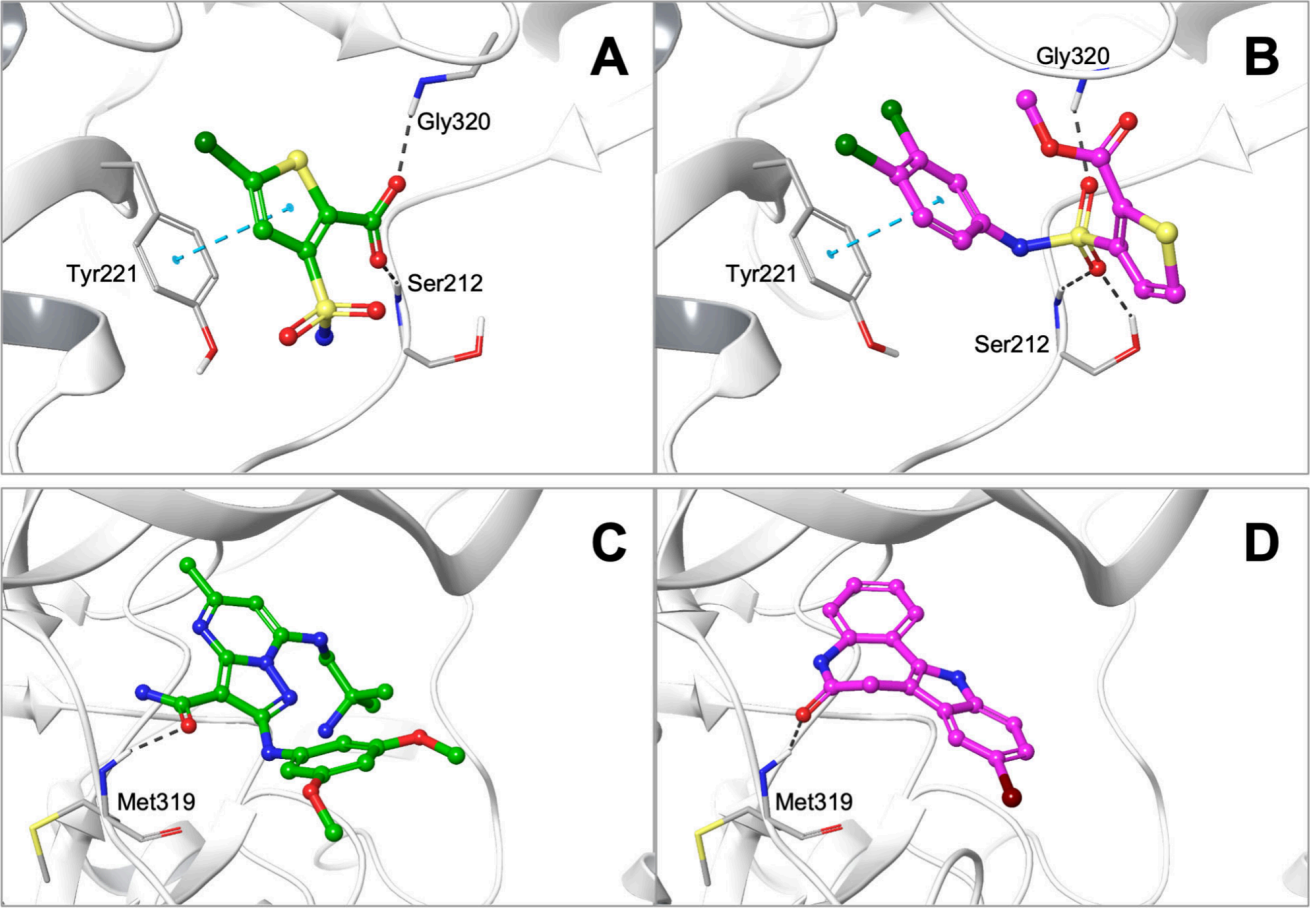
Examples of (A, C) cocrystallized ligand poses and (B, D) ligand poses generated with DiffDock-L for compounds that are structurally distinct from any cocrystallized ligand. Despite their structural differences, the pairs of molecules display consistent protein–ligand interaction patterns (quantified by PLIFs). (A) Crystal structure of AmpC ß-lactamase in complex with the fragment-sized inhibitor 5-chloro-3-sulfamoylthiophene-2-carboxylic acid (PDB 4KZ3). (B) Docking pose generated with DiffDock-L for a known AmpC ß-lactamase inhibitor, methyl 3-[(3,4-dichlorophenyl)­sulfamoyl]-2-thiophenecarboxylate. (C) Crystal structure of leukocyte-specific protein tyrosine kinase (LCK) in complex with a pyrazolo pyrimidine-based inhibitor (PDB 3AC2). (D) Docking pose generated with DiffDock-L for kenpaullone, an inhibitor of several kinases including LCK. Dashed lines indicate hydrogen bonds and aromatic interactions (note that PLIFs account for the full spectrum of protein–ligand interactions, including hydrophobic interactions, which are not illustrated in this figure).

### Computational Performance

The computational performance of the docking algorithms was tested on a workstation equipped with an AMD EPYC 7713 64-Core processor, 258 GB of RAM, and a 24GB NVIDIA GeForce RTX 3090 graphics card. On this machine and GPU, the average runtime across the 43 DUDE-Z targets was 62.29 ± 13.65 s/compound for DiffDock-L (Figure S17). In comparison, the average runtime for Vina was 4.95 ± 2.61 s/compound on the CPU (note that these results reflect the pose generation process and do not account for the CPU time used for preparing the protein structure, the binding pocket, and the small molecules).

The runtime of DiffDock-L showed a strong correlation (Pearson r = 0.85, *p*-value < 10^–4^) with the size of the proteins (indicated by the number of residues in the structures), while the runtime of Vina exhibited a strong correlation (Pearson r = 0.92, *p*-value < 10^–4^) with the size of the small molecules (indicated by the average MW of compounds for each target; Figure S18).

The fact that both pose sampling methods use distinct hardware components impedes the direct comparison of the computational efficiency. Note also that DiffDock-L, by design, performs blind docking, taking the full protein structure into account. In contrast, Vina focuses on a specified binding pocket.

## Conclusions

This study presents a framework that integrates ML-based pose sampling using DiffDock-L with the Vina, Gnina, and RTMScore scoring functions. We evaluated the VS performance of the various combinations of docking and scoring functions, the complementarity of ML-based and physics-based pose sampling, and DiffDock’s sampling capability in cross-docking scenarios.

Our findings demonstrate that ML-based pose sampling performs comparably to physics-based pose sampling methods in VS applications. Moreover, the choice of scoring function can be crucial to the success of VS. By employing various evaluation criteria, we show that the poses predicted by ML-based methods are physically, chemically, and biologically relevant. Furthermore, ML-based pose sampling effectively models viable poses for structurally unrelated active compounds. However, integrating ML-based pose sampling with the physics-based approach did not significantly enhance VS success.

While benchmarking on the DUDE-Z provides valuable insights across a range of targets and types of small molecules, the study underscores the need for a more comprehensive evaluation, particularly addressing the generalizability of the ML-based pose sampling methods for VS across unseen targets. Future research should focus on curating more diverse datasets that include targets substantially different from the current training data. Such expansive datasets would enable more robust statistical analysis and a deeper understanding of the performance variations in ML-based VS methods.

## Supplementary Material



## Data Availability

All data used in this work are part of DUDE-Z, which can be accessed at https://dudez.docking.org/. The source code for the analyses presented in this work is available at https://github.com/lan-codes/Benchmark_VS.

## Notes

Additional details on parameters used in executing the docking programs, statistics of the processed molecules, correlation analyses for docking scores, details on rescoring with RTMScore, performance of the methods on different chemical spaces, correlations between the methods’ performance and protein binding site properties, data curation process and the methods’ performance on the new VS test set, validity and plausibility analyses of docking poses, statistics on protein–ligand interaction profiles of the docking poses and reference ligands for individual targets, and the runtime details of the methods (PDF)
