# Supplementary material for: Integrating Machine Learning-Based Pose Sampling with Established Scoring Functions for Virtual Screening
Source: J Chem Inf Model. 2025 May 9;65(10):4833–43. doi: 10.1021/acs.jcim.5c00380 (PMC12117556; doi:10.1021/acs.jcim.5c00380)
Supplement: Supplementary file 1 [file ci5c00380_si_001.pdf]

Supporting Information

Integrating Machine Learning-Based Pose Sampling with Established Scoring Functions for Virtual Screening

Thi Ngoc Lan Vu[1,2,3], Hosein Fooladi[1,2,3] and Johannes Kirchmair[1,2]

[1] Department of Pharmaceutical Sciences, Division of Pharmaceutical Chemistry, Faculty of Life Sciences, University of Vienna, Josef-Holaubek-Platz 2, 1090 Vienna, Austria

[2] Christian Doppler Laboratory for Molecular Informatics in the Biosciences, Department for Pharmaceutical Sciences, University of Vienna, Josef-Holaubek-Platz 2, 1090 Vienna, Austria

[3] Vienna Doctoral School of Pharmaceutical, Nutritional and Sport Sciences (PhaNuSpo), University of Vienna, Josef-Holaubek-Platz 2, 1090 Vienna, Austria

* Corresponding author: johannes.kirchmair@univie.ac.at

Figures

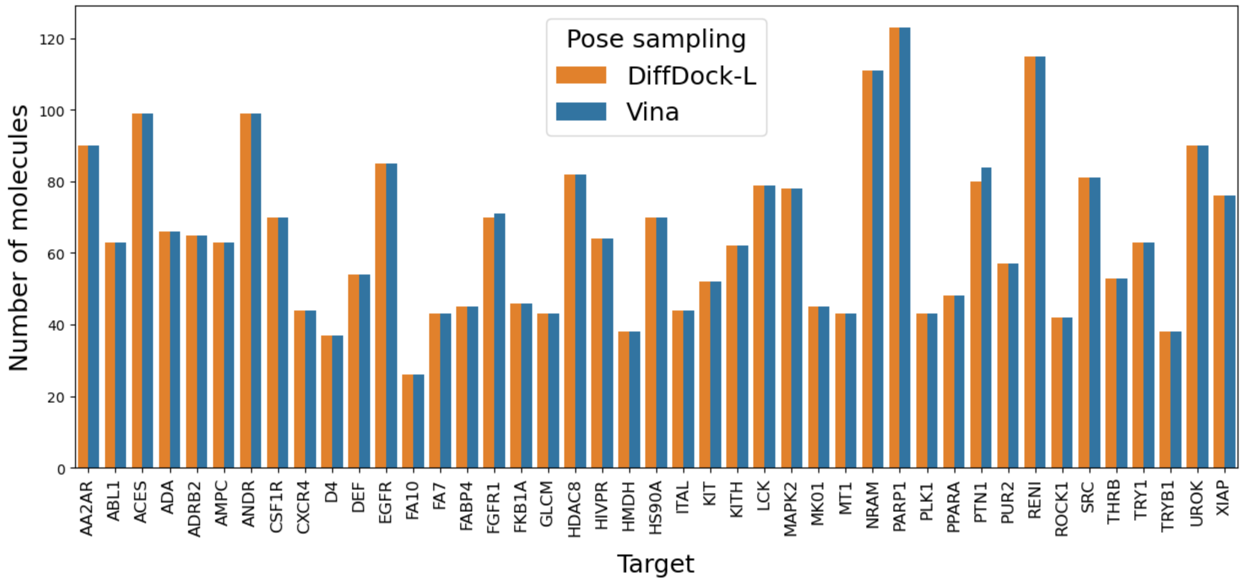

Figure S1. Number of active compounds successfully processed with each pose sampling method.

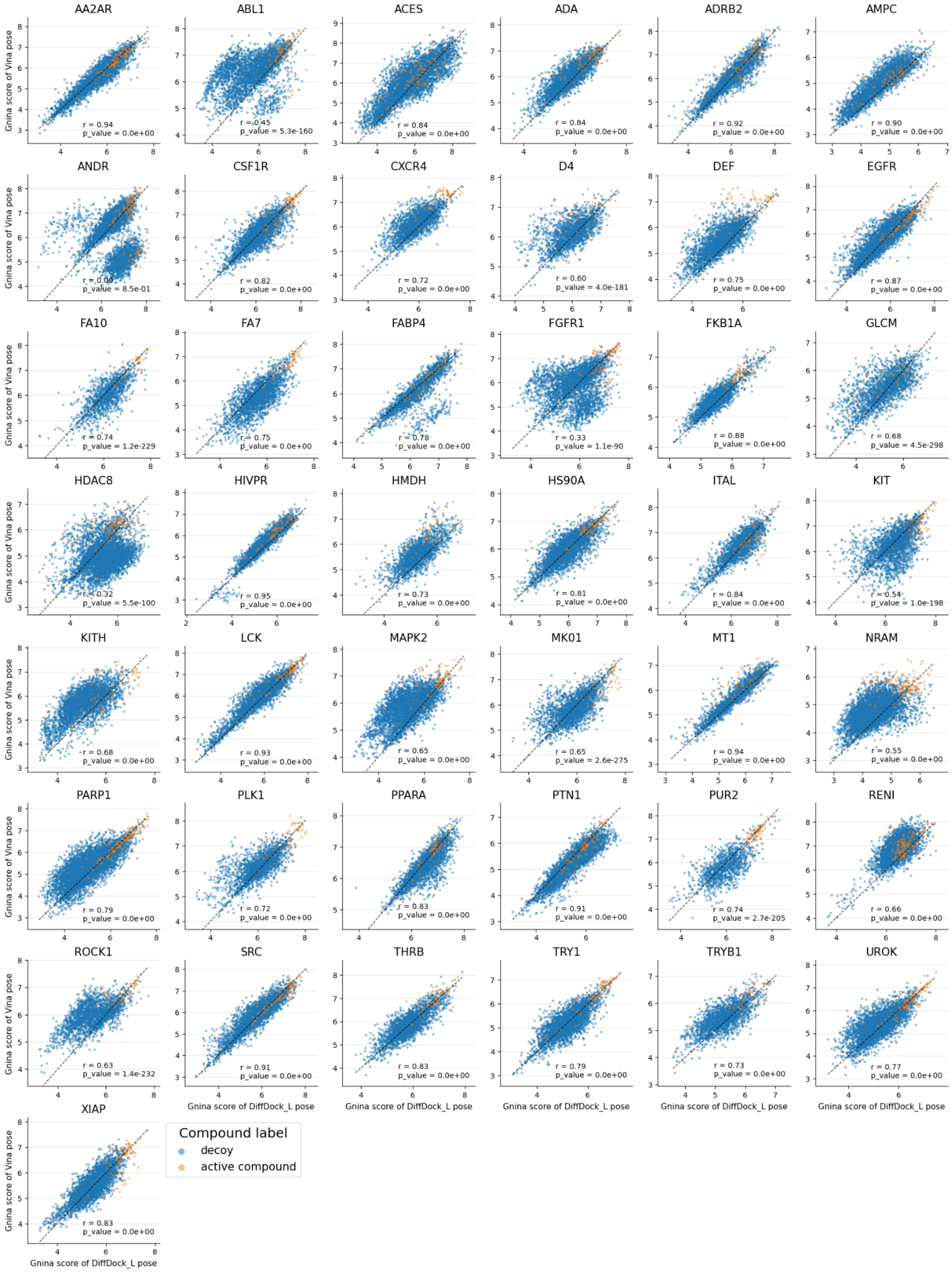

Figure S2. Correlations between Gnina scores of Vina poses and DiffDock-L poses for individual molecules.

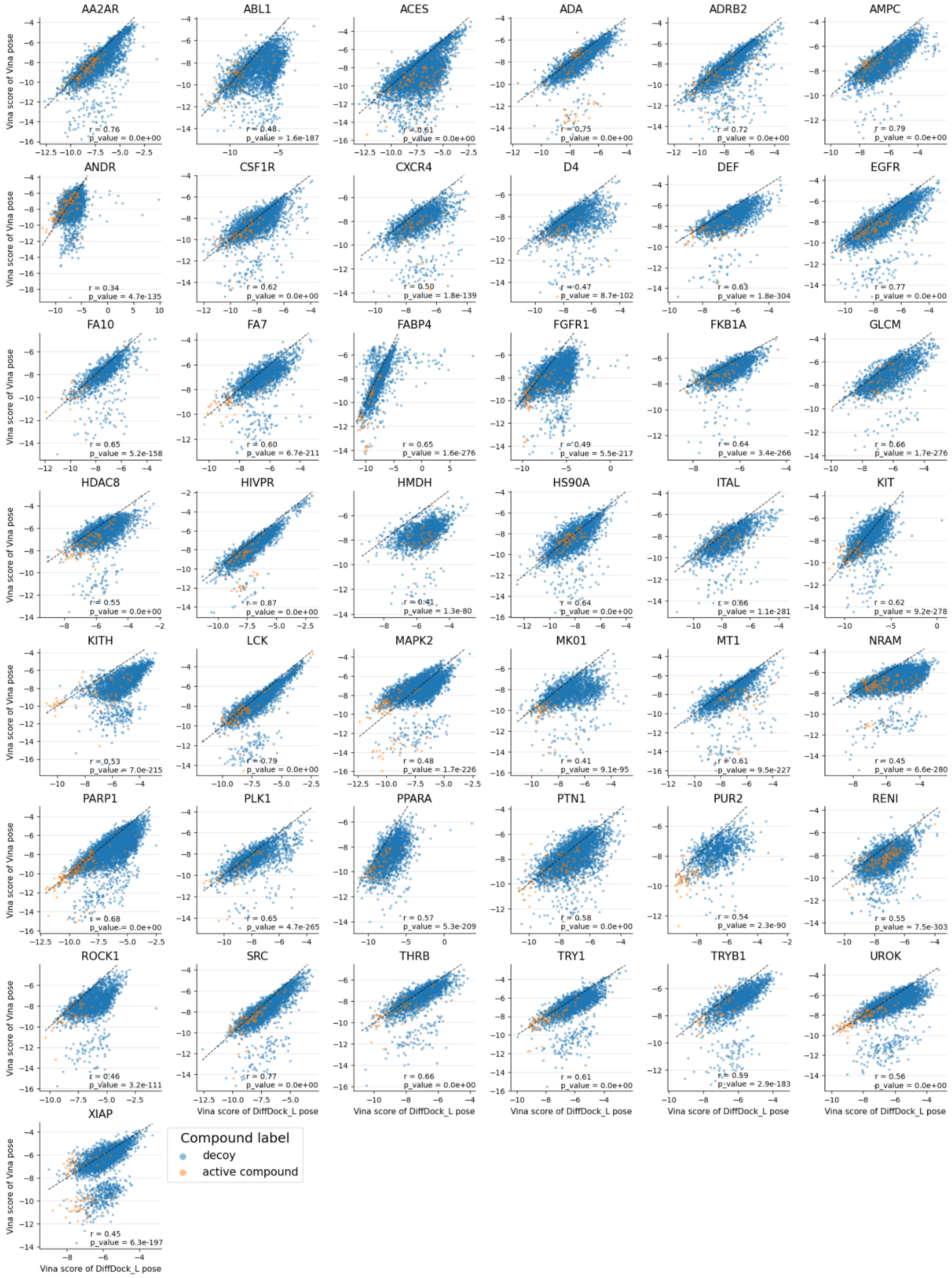

Figure S3. Correlations between Vina scores of Vina poses and DiffDock-L poses for individual molecules.

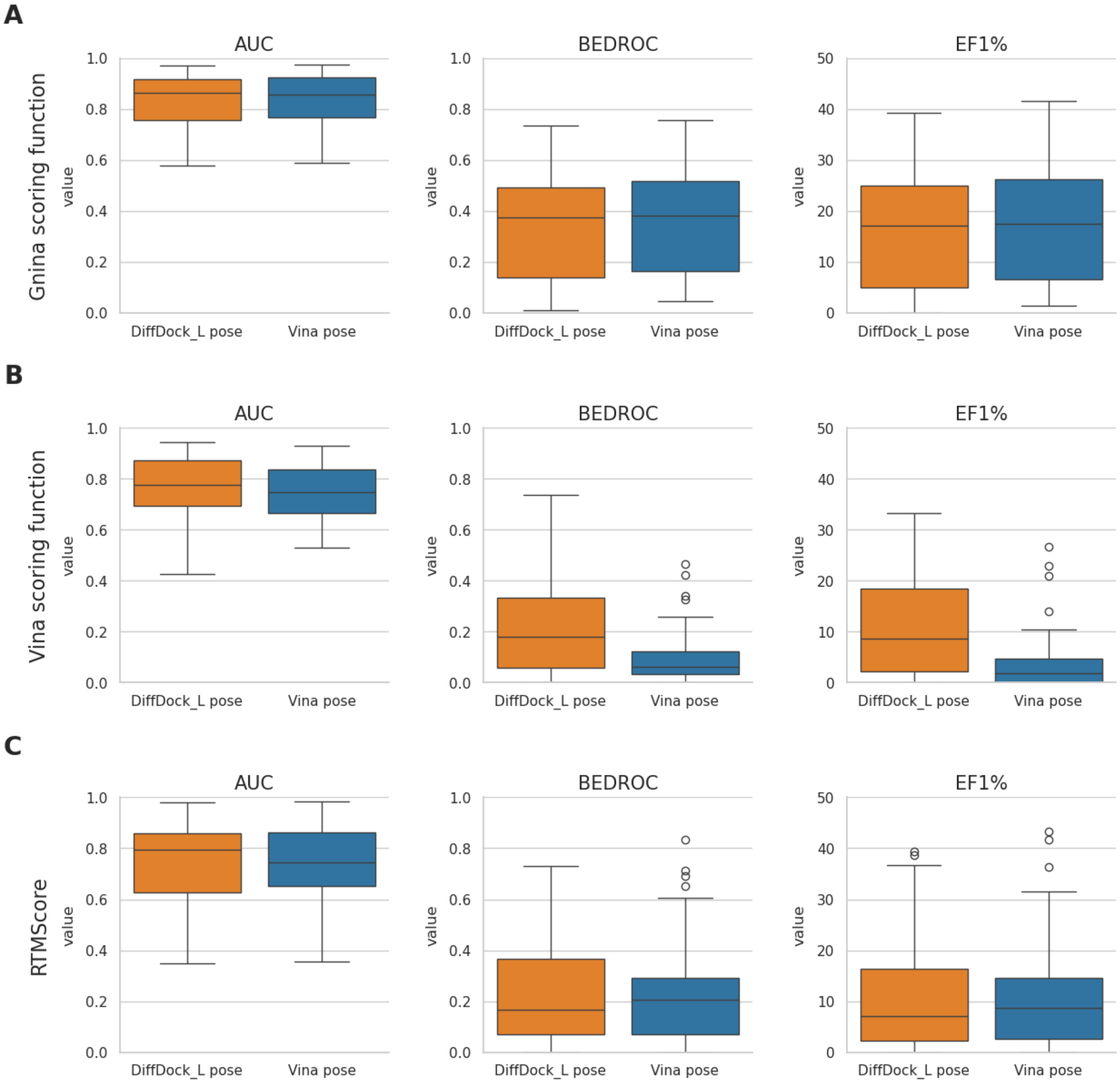

Figure S4. VS performance on the DUDE-Z dataset using the DiffDock-L pose sampling method (orange boxes) and the Vina pose sampling method (blue boxes) combined with (A) the Gnina scoring function, (B) the Vina scoring function, and (C) the RTMScore.

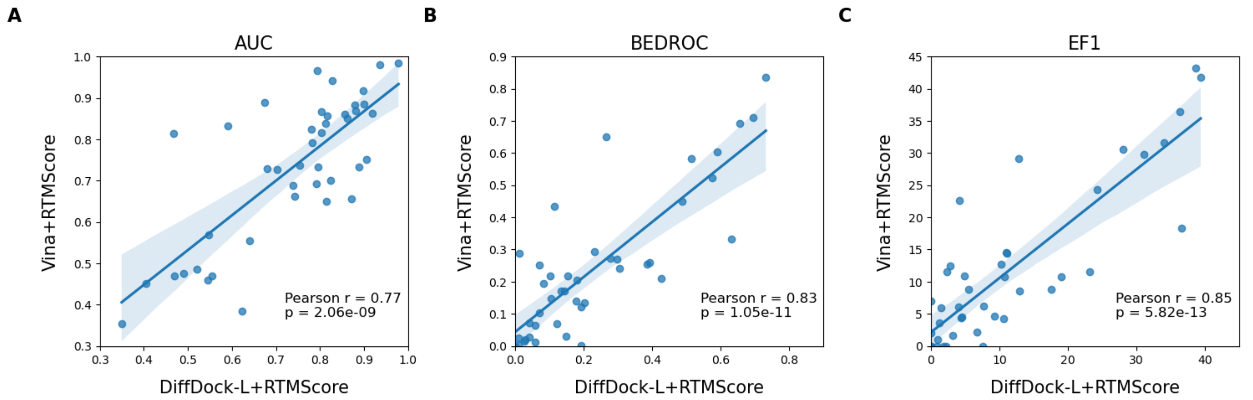

Figure S5. Correlations between the AUC, EF1%, and BEDROC scores on DUDE-Z targets for DiffDock-L and Vina, both combined with the RTMScore.

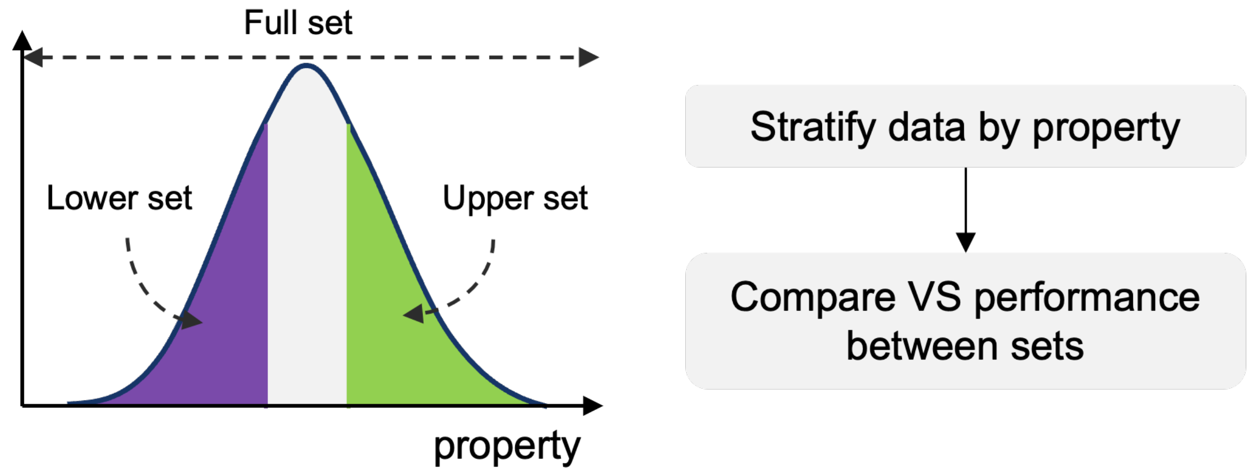

Figure S6. Data stratification for chemical space analysis.

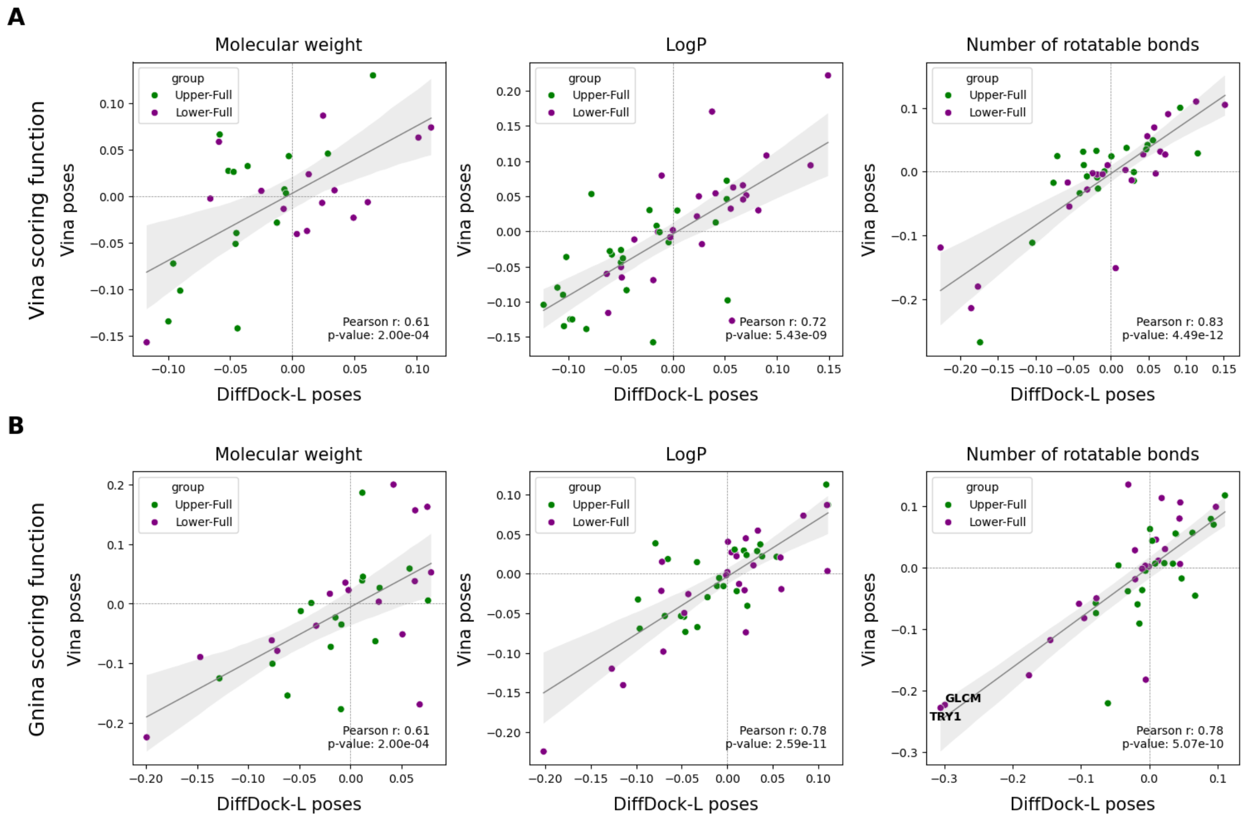

Figure S7. Correlations between the changes in AUC scores of DiffDock-L and Vina when moving from full set to the upper (green) and lower (purple) sets according to each property.

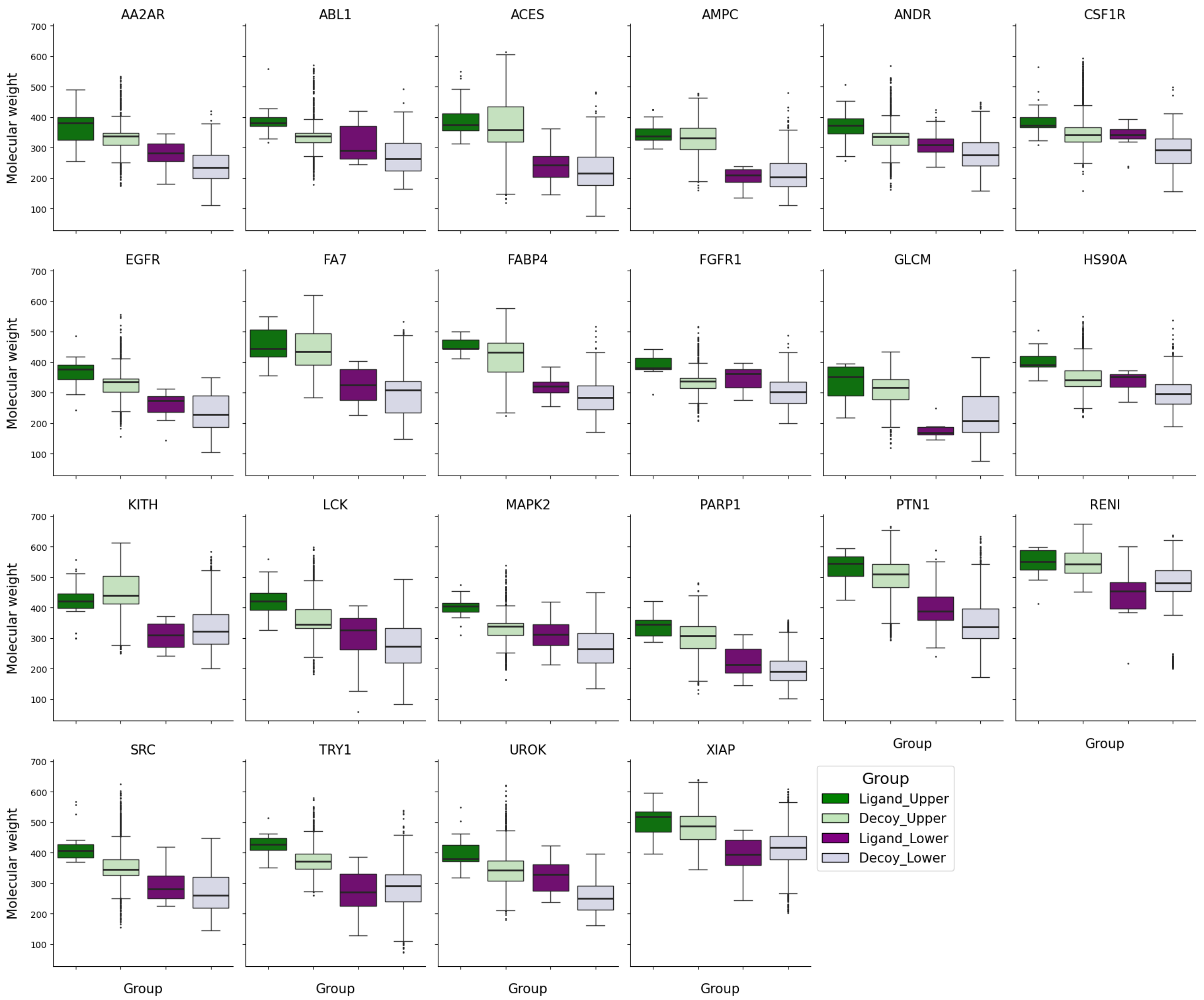

Figure S8. Distribution of molecular weight of the compounds in each group stratified by the number of RBs.

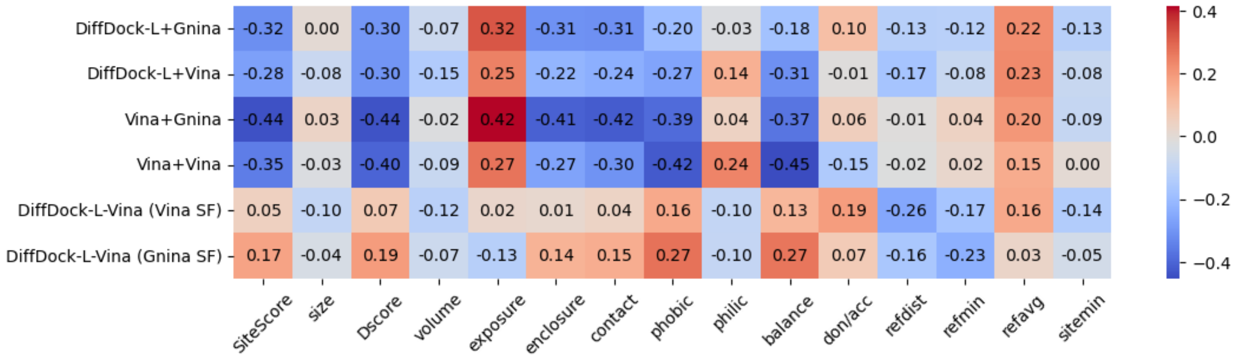

Figure S9. Pearson correlation coefficients between protein binding site properties and the performance of the methods.

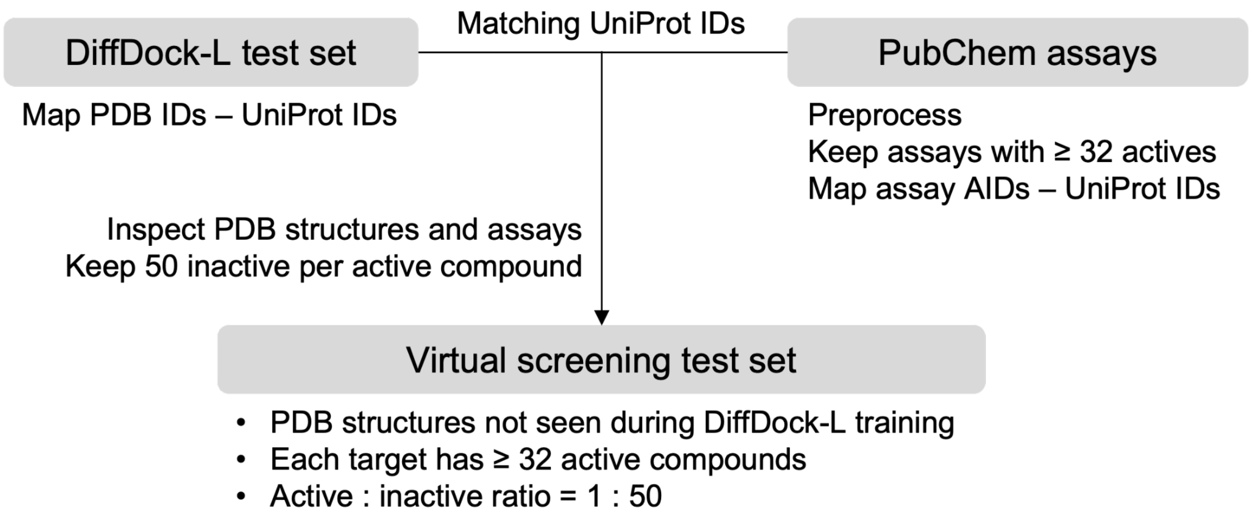

Figure S10. Curation process of the new VS test set with protein structures distinct from any protein structures in the DiffDock-L training data. Each of the selected targets has a sufficient number of active compounds.

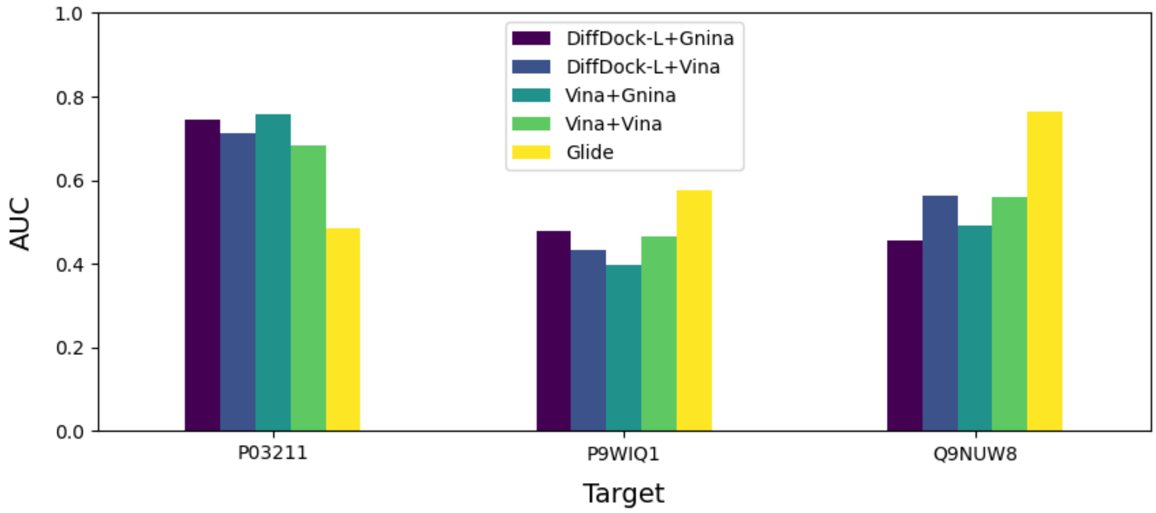

Figure S11. AUC scores of the VS setups on three targets not included in the DiffDock-L training data. The performance of Glide docking is shown for reference.

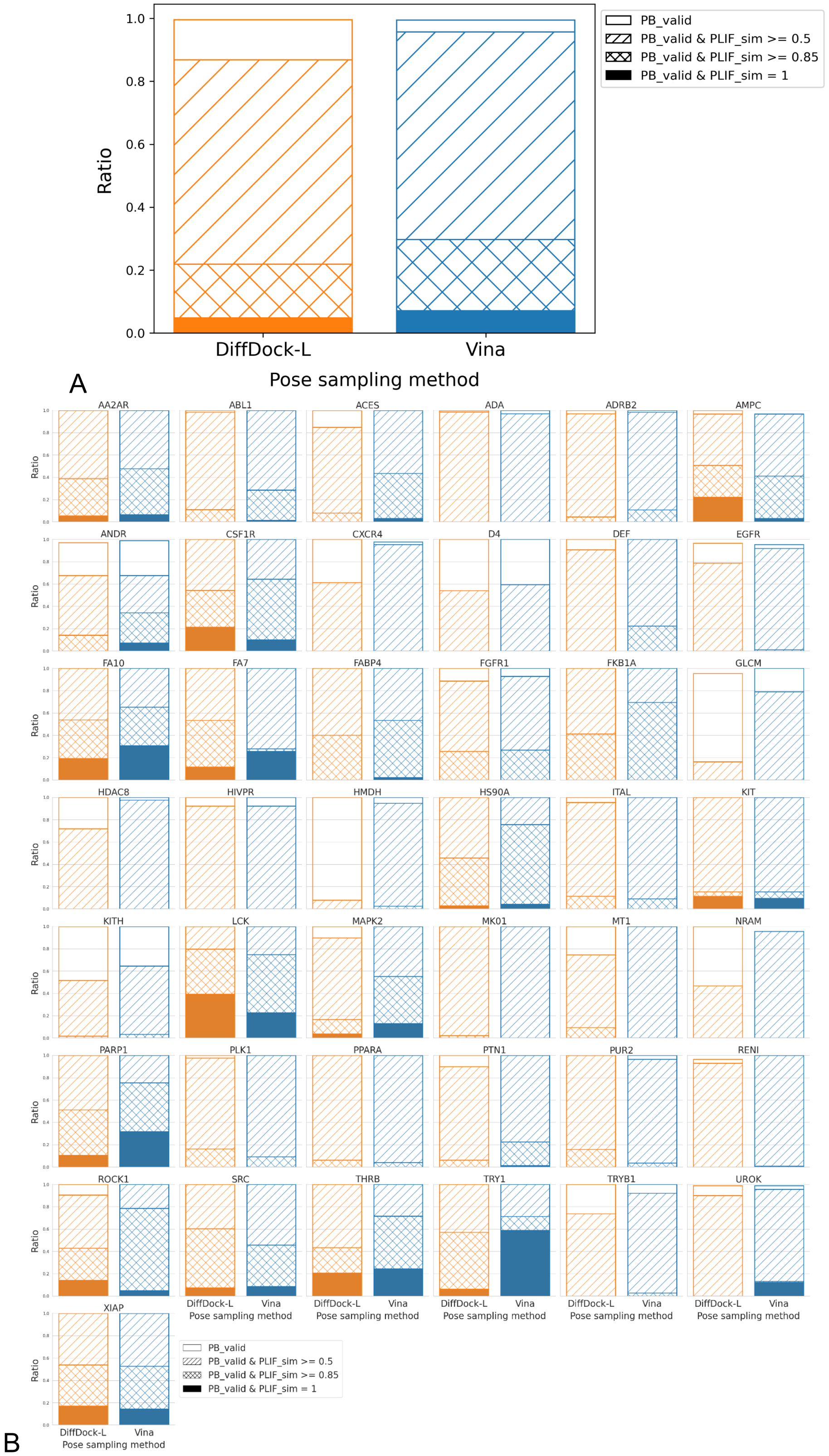

Figure S12. Percentage of active molecules across (A) all targets and (B) the individual DUDE-Z targets having at least one pose by each pose sampling method passing the physicochemical plausibility check (PB_valid) and relevance assessment (PLIF_sim).

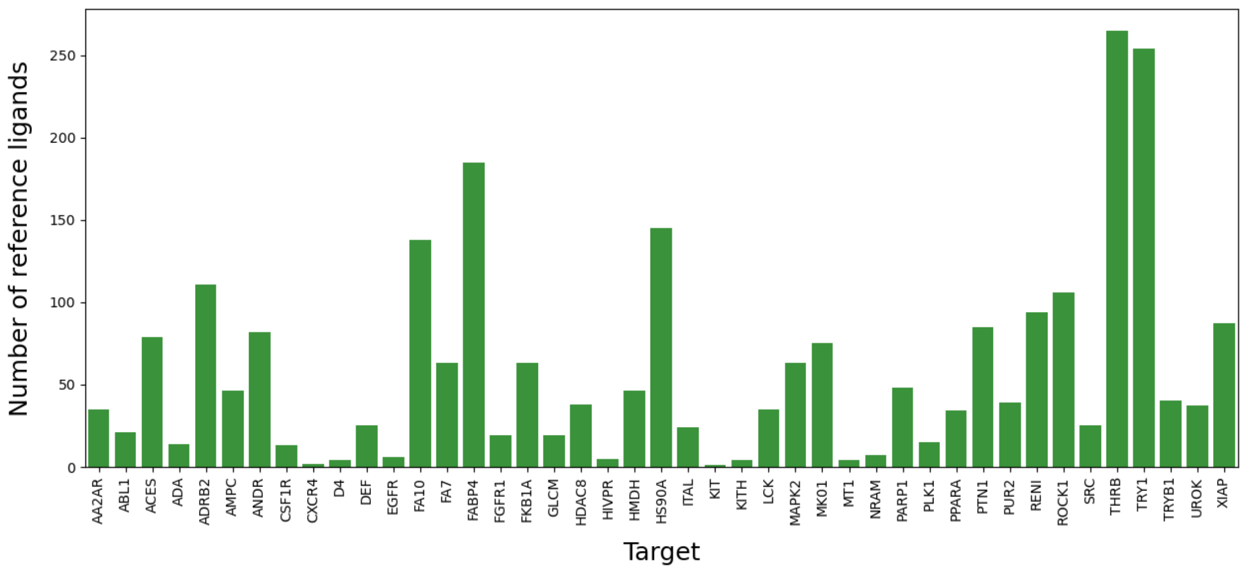

Figure S13. Number of protein-ligand complexes retrieved from the PDB for each DUDE-Z target.

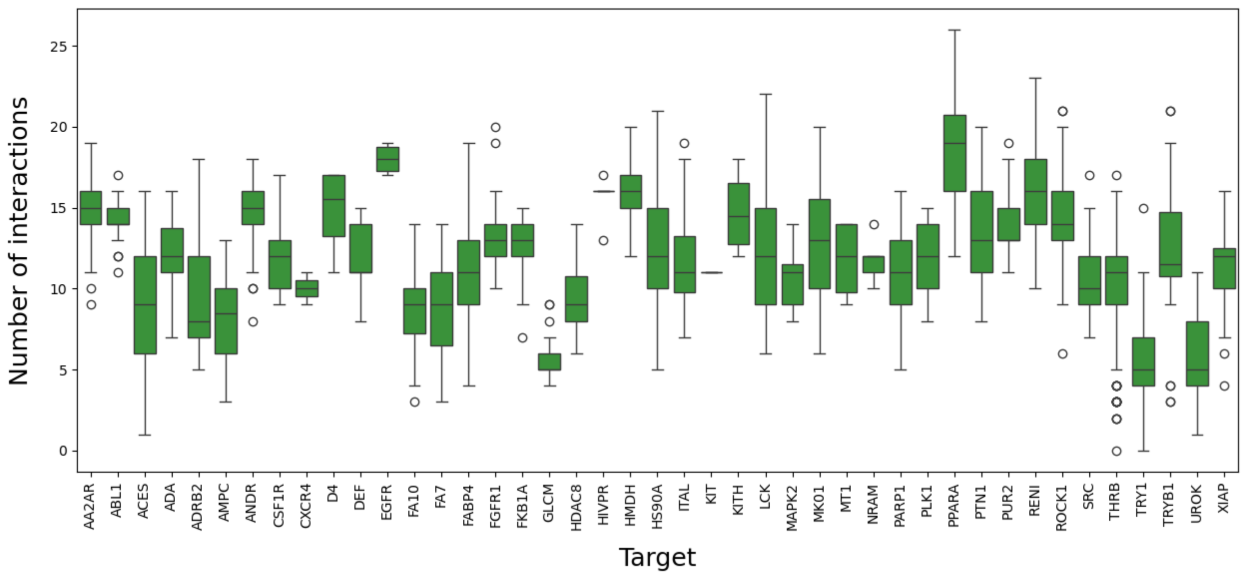

Figure S14. Distribution of protein-ligand interactions of reference ligands retrieved for each DUDE-Z target.

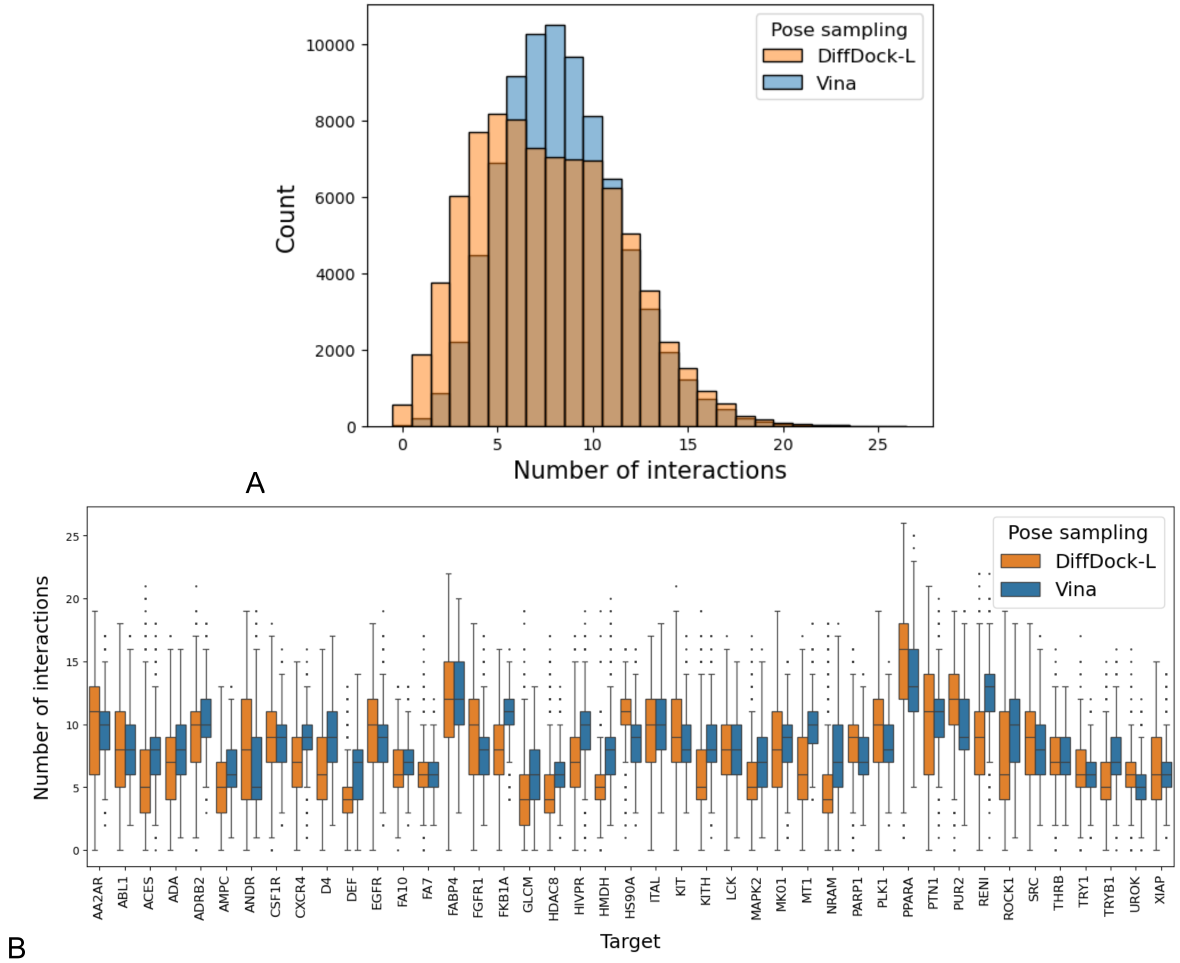

Figure S15. Distribution of the number of protein-ligand interactions of poses sampled by either pose sampling method for active molecules across (A) all and (B) individual DUDE-Z targets.

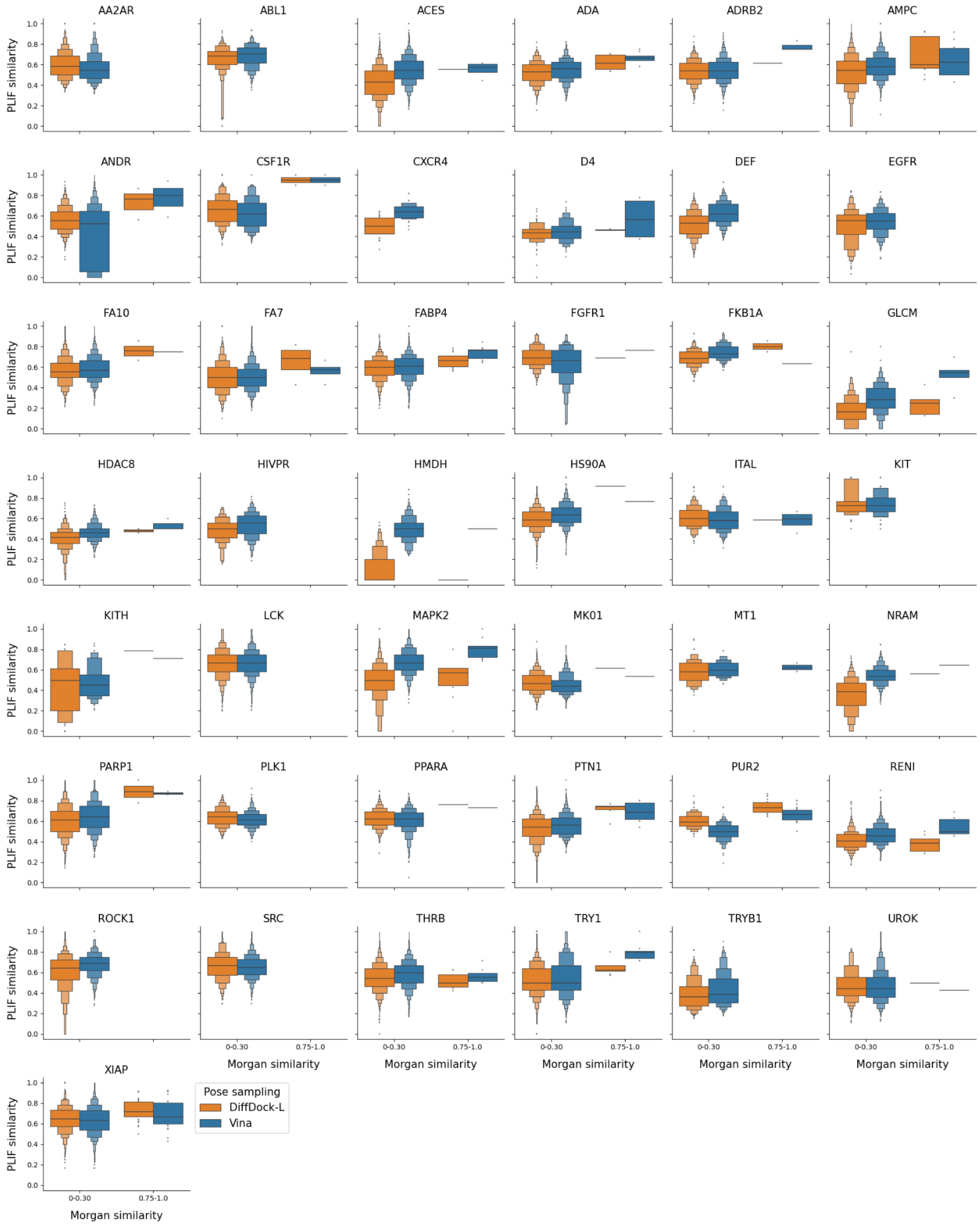

Figure S16. PLIF similarities between pairs of (A) structurally distinct measured and predicted protein-ligand complexes (Tanimoto coefficient based on Morgan2 fingerprints not exceeding 0.30) and (B) structurally related measured and predicted protein-ligand complexes (Tanimoto coefficient greater than 0.75) sampled with DiffDock-L and Vina for the individual DUDE-Z targets.

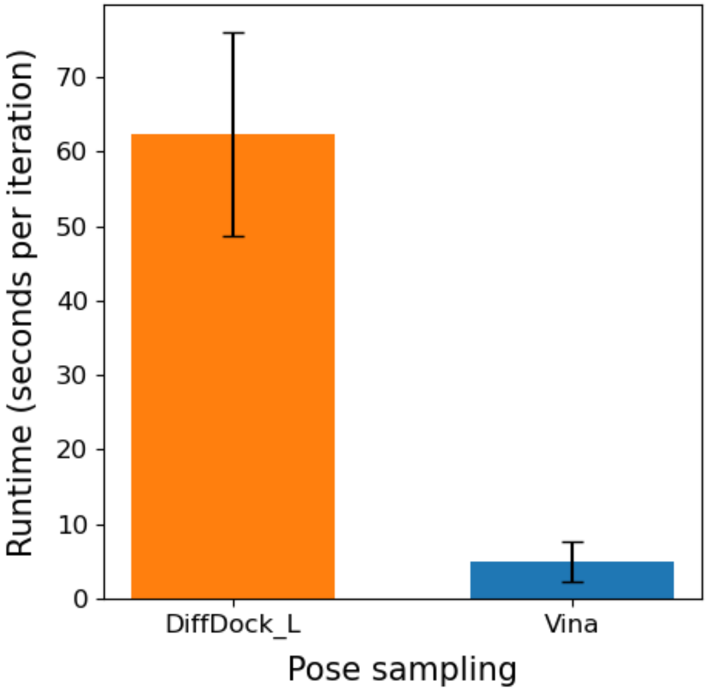

Figure S17. Average runtimes for pose sampling with DiffDock-L and Vina for the 43 DUDE-Z targets.

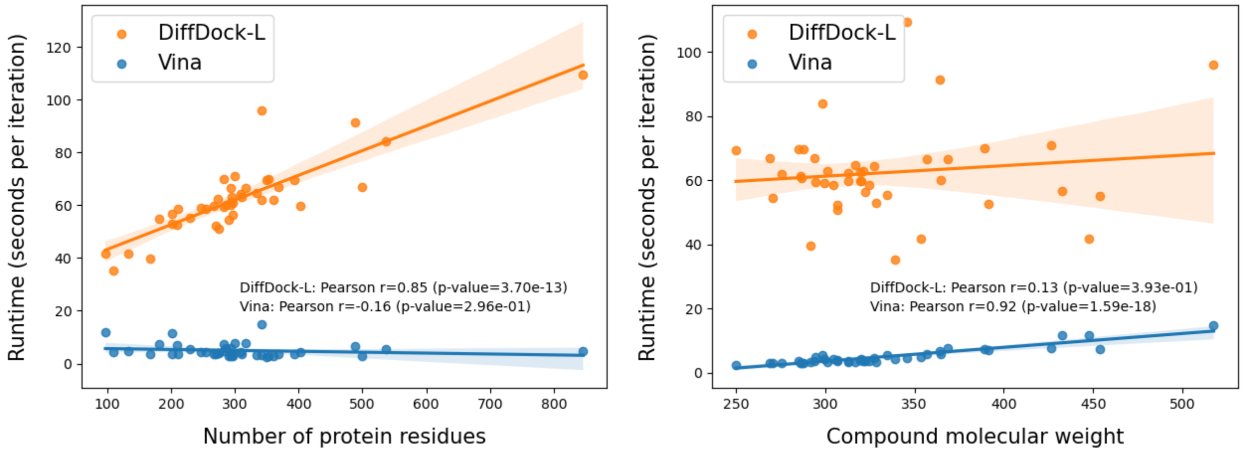

Figure S18. Correlations between DiffDock-L and Vina runtimes and (A) the number of residues in a protein structure and (B) the MW of the docked small molecules.

Tables

Table S1. The parameters used in executing DiffDock-L and Vina programs.

DiffDock-L parameters

- samples_per_complex: 30
- actual_steps: 19
- different_schedules: false
- inf_sched_alpha: 1
- inf_sched_beta: 1
- inference_steps: 20
- limit_failures: 5
- no_final_step_noise: true
- no_model: false
- no_random: false
- no_random_pocket: false
- ode: false
- old_filtering_model: true
- old_score_model: false
- resample_rdkit: false
- sigma_schedule: expbeta
- initial_noise_std_proportion: 1.4601642460337794
- temp_psi_rot: 0.9022615585677628
- temp_psi_tor: 0.5946212391366862
- temp_psi_tr: 0.727287304570729
- temp_sampling_rot: 2.06391612594481
- temp_sampling_tor: 7.044261621607846
- temp_sampling_tr: 1.170050527854316
- temp_sigma_data_rot: 0.7464326999906034
- temp_sigma_data_tor: 0.6943254174849822
- temp_sigma_data_tr: 0.9299802531572672

Vina parameters

- sf_name: 'vina'
- box_size: [30,30,30]
- exhaustiveness: 32
- n_poses: 30
- min_rmsd: 1.0
- max_evals: 0
- energy_range: 3.0
- coordinates_only: False

Table S2. Protein binding site properties of DUDE-Z targets.[1]

Target	SiteScore	size	Dscore	volume	exposure	enclosure	contact	phobic	philic	balance	don/acc	refdist	refmin	refavg	sitemin
AA2AR	0.96	747	1.08	633.52	0.71	0.45	0.44	0.70	0.41	1.70	1.35	2.59	0.25	0.65	0.89
ABL1	0.94	266	1.04	210.60	0.67	0.48	0.49	0.75	0.59	1.27	1.31	2.67	0.14	0.55	0.61
ACES	1.03	396	1.11	451.39	0.54	0.64	0.70	0.68	0.67	1.02	0.82	6.38	0.15	0.59	4.23
ADA	0.98	542	1.08	502.84	0.63	0.52	0.51	0.42	0.54	0.79	1.08	3.75	0.25	0.62	1.57
ADRB2	1.02	791	1.09	809.14	0.44	0.63	0.72	0.81	0.69	1.18	0.79	8.02	0.23	0.66	3.06
AMPC	0.93	627	1.00	611.23	0.66	0.51	0.55	0.18	0.75	0.25	0.65	2.90	0.11	0.57	0.94
ANDR	1.29	154	1.36	178.02	0.19	0.97	1.16	3.57	0.49	7.31	0.52	0.63	0.06	0.60	0.69
CSF1R	0.95	465	1.05	404.74	0.67	0.49	0.51	0.45	0.58	0.77	1.02	4.45	0.31	0.87	0.98
CXCR4	0.97	1057	1.05	1054.04	0.58	0.56	0.55	0.43	0.69	0.63	1.39	5.16	0.20	0.66	2.29
D4	1.01	620	1.08	573.84	0.45	0.62	0.67	0.68	0.72	0.94	1.29	6.54	0.20	0.62	3.58
DEF	0.91	494	0.99	381.42	0.68	0.46	0.45	0.26	0.69	0.37	1.03	3.71	0.24	0.65	0.51
EGFR	0.97	603	1.04	546.40	0.53	0.55	0.59	0.64	0.70	0.91	1.39	4.90	0.11	0.57	1.44
FA10	0.92	505	1.04	426.35	0.73	0.43	0.43	0.36	0.49	0.73	0.92	4.39	0.14	0.62	2.50
FA7	0.89	586	0.98	401.65	0.73	0.41	0.40	0.09	0.64	0.14	0.90	1.99	0.19	0.75	1.45
FABP4	1.20	340	1.24	378.33	0.14	0.93	0.96	1.31	0.81	1.62	0.29	0.84	0.11	0.54	0.98
FGFR1	0.96	728	1.02	734.71	0.60	0.57	0.59	0.35	0.80	0.44	0.70	7.46	0.26	0.61	5.33
FKB1A	0.93	200	1.05	143.37	0.77	0.41	0.36	0.31	0.41	0.74	0.73	3.13	0.09	1.11	1.08
GLCM	0.97	409	1.05	349.52	0.60	0.53	0.53	0.34	0.63	0.54	1.15	5.43	0.26	0.69	2.01
HDAC8	0.92	170	1.03	166.01	0.78	0.42	0.41	0.24	0.49	0.48	0.60	3.45	0.30	1.06	0.85
HIVPR	0.95	822	1.04	638.32	0.64	0.48	0.45	0.36	0.56	0.64	1.01	0.99	0.18	0.62	1.15
HMDH	0.96	1062	1.04	991.61	0.62	0.53	0.54	0.31	0.65	0.48	0.67	3.96	0.09	0.62	2.25
HS90A	0.97	527	1.05	469.57	0.59	0.53	0.57	0.51	0.62	0.83	1.12	3.35	0.25	0.58	2.13
ITAL	0.94	389	1.07	249.02	0.72	0.42	0.38	0.60	0.37	1.61	0.97	1.84	0.16	0.75	0.63
KIT	0.96	365	1.05	322.76	0.67	0.51	0.50	0.36	0.59	0.60	0.62	4.45	0.07	0.55	0.92
KITH	0.94	750	1.02	668.85	0.59	0.52	0.52	0.23	0.70	0.33	0.60	6.01	0.24	0.62	1.63
LCK	0.96	586	1.05	389.31	0.63	0.52	0.48	0.33	0.62	0.53	1.79	3.07	0.12	0.65	1.06
MAPK2	0.94	661	1.02	498.72	0.61	0.50	0.50	0.27	0.69	0.39	0.86	1.49	0.12	0.58	0.66
MK01	0.95	774	1.05	645.53	0.69	0.48	0.47	0.32	0.55	0.58	0.93	3.17	0.12	0.60	1.11
MT1	1.05	301	1.17	312.47	0.58	0.57	0.61	1.31	0.36	3.60	0.63	4.73	0.26	0.53	1.22

NRAM	0.93	396	1.01	332.71	0.66	0.51	0.48	0.11	0.74	0.15	0.52	4.96	0.07	0.63	1.17
PARP1	0.97	803	1.06	768.66	0.64	0.53	0.53	0.34	0.62	0.54	0.76	2.47	0.18	0.61	2.16
PLK1	0.96	765	1.04	651.36	0.53	0.54	0.57	0.37	0.68	0.54	0.61	5.82	0.17	0.64	1.89
PPARA	0.98	785	1.08	750.83	0.61	0.54	0.57	0.73	0.57	1.29	1.03	5.59	0.17	0.58	3.01
PTN1	0.89	347	0.96	265.48	0.72	0.45	0.45	0.11	0.76	0.15	0.54	5.65	0.22	0.56	1.66
PUR2	0.93	445	1.00	396.17	0.64	0.50	0.51	0.17	0.75	0.23	0.44	6.45	0.21	0.77	2.31
RENI	1.02	968	1.11	840.69	0.51	0.58	0.58	0.62	0.55	1.13	1.24	4.68	0.17	0.62	1.89
ROCK1	0.97	649	1.04	696.63	0.61	0.56	0.55	0.37	0.73	0.50	1.63	3.16	0.15	0.58	1.70
SRC	1.00	672	1.11	590.30	0.61	0.55	0.55	0.55	0.49	1.12	1.28	4.22	0.24	0.59	4.05
THRB	0.94	643	1.04	566.64	0.64	0.49	0.52	0.35	0.60	0.58	1.01	3.75	0.12	0.62	1.13
TRY1	0.91	442	1.02	285.38	0.76	0.40	0.40	0.24	0.49	0.49	0.83	1.88	0.18	0.95	0.91
TRYB1	0.97	796	1.07	699.21	0.69	0.51	0.51	0.33	0.56	0.59	1.13	5.01	0.18	0.66	2.45
UROK	0.91	427	1.00	357.75	0.72	0.46	0.45	0.14	0.65	0.21	1.01	2.67	0.22	0.67	1.22
XIAP	0.87	186	0.99	112.16	0.84	0.34	0.30	0.11	0.46	0.23	1.22	4.44	0.22	2.02	1.38

[1] Column names:

SiteScore: How well the site can bind to ligands
Dscore: How druggable the site is
size: Number of site points enclosed within the site
volume: The site's volume ($Å^3$)
exposure, *enclosure*: The degree of binding site's accessibility to solvent molecules
contact: Quantifies the strength of van der Waals interactions between a probe at an average site point and the protein
phobic, philic, balance: The site's hydrophobic and hydrophilic characteristics, and the ratio between the two properties.
don/acc: The hydrogen bond directionality potential of an optimally structured ligand at the site (donor vs acceptor tendency)
refdist: The distance between the centroid of the site and the centroid of the reference ligand
refmin: The minimum distance between any site point and any reference atom
refavg: The average value of the shortest distances between the reference ligand atoms and any site points
sitemin: The shortest distance between the site-point centroid and any reference atom

Table S3. Information on the targets included in the new virtual screening test set.

	P03211	P9WIQ1	Q9NUW8
Protein	Epstein-Barr nuclear antigen 1 (EBNA1)	UDP-galactopyranose mutase	Tyrosyl-DNA phosphodiesterase 1
PDB ID (crystal-ligand ID)	6NPP (KWG)	4RPJ (UDP)	6N19 (K8V)
Crystal complex	Complex with an inhibitor	Complex with substrate UDP	Inhibitor at catalytic site of TDP1
ECOD annotation	Viral DNA-binding domain	Nucleotide-binding domain	Phospholipase D/nuclease
Selected Pubchem AID	1950 - 2381 EBNA1 inhibitors	504406 - 540359 UDP competing inhibitors	485290 - 489007 TDP1 inhibitors
# actives	61	79	277

Exploring the RTMScore scoring function

The poses generated by Vina pose sampling and the minimized poses sampled with DiffDock-L were loaded together with the respective protein structures to the RTMScore. Rescoring was performed using the "rtmscore.py" provided in the RTMScore github repository under the "example" folder (https://github.com/sc8668/RTMScore/tree/main/example), utilizing the script's parameters as listed below:

Command:

```
python rtmscore.py -p protein_path -l ligand_path -o out_prefix
```

Parameters:

- dist_threhold: 5
- batch_size: 128
- num_workers: 10
- num_node_featsp: 41
- num_node_featsl: 41
- n_gaussians: 10
- num_edge_featsp: 5
- num_edge_featsl: 10
- hidden_dim0: 128
- hidden_dim: 128
- dropout_rate: 0.10

To account for the various locations of DiffDock-L poses (DiffDock-L blind docks without prior knowledge of binding pocket) and ensure time efficiency, the whole protein structures were used to generate pocket graphs. This should not affect RTMScore evaluations since the scoring function considers only the residues within 5 Å from the processed pose (dist_threshold=5). The option helped boost the time compared to generating individual pocket graphs for each pose (about 200 times faster). The option does, however, require more memory, especially for large proteins. Indeed, of all 43 DUDE-Z targets, the protein HMDH with 846 residues was not loadable

by our GPU with 24GB of memory. The results reported in Figure S4 are for 42 DUDE-Z targets; HMDH is omitted due to the unsuccessful loading of the protein structures to RTMScore.

In general, DiffDock-L showed comparable AUC scores on DUDE-Z targets when paired with RTMScore compared to Gnina and Vina scoring functions. The early enrichment metrics (BEDROC and EF1%) also demonstrated similar ranges to the Vina scoring function and slightly lower values than the Gnina scoring function. In additions, the AUC scores achieved by DiffDock-L and Vina when paired with RTMScore showed strong correlations (Peason r = 0.77, p-value < 10^{-4}, Figure S5A), consistent with the results observed when the two pose sampling methods were paired with Vina and Gnina scoring functions (Figure 4, main text). Furthermore, the high correlations were also observed for BEDROC and EF1% achieved by DiffDock-L and Vina when paired with RTMScore (Peason r = 0.85 and 0.83 respectively, p-value < 10^{-4}, Figure S5B, C), similar to the correlations observed with the Gnina scoring function (as detailed in the main text). Overall, the observations further underscore the substantial influence of scoring functions on VS performance. These results encourage further explorations and applications of RTMScore and other available scoring functions for incorporation with ML-based pose sampling into VS workflow.

Binding site properties analysis

Methods

The binding site surrounding the crystal ligand denoted in DUDE-Z for each target was analyzed using the "Binding Site Detection" function of SiteMap. The option was set to "Evaluate a single binding site region" within a buffer region of 10 Å around the crystal ligand. The mode was set to "Detect shallow binding site" to adapt to shallow binding sites in the proteins (e.g., HMDH and TRYB1). Other parameters were maintained as default values. The binding sites identified by SiteMap were manually reviewed, taking into account the location, coverage, and SiteScore values to select the most suitable binding site for each target.

Results

A total of 15 properties for each binding site were calculated, indicating the volume ("size", "volume"), solvent exposure ("exposure", "enclosure"), contact with the receptor ("contact"), hydrophobicity characteristics ("phobic", "philic", "balance"), hydrogen bond tendency of an optimal ligand for the site ("don/acc"), and the relative location and coverage with the reference ligand ("refdist", "refmin", "refavg", "sitemin"). These reported properties are presented in Table S2. The correlations between these properties and the performance of DiffDock-L and Vina across different setups were analyzed. No significant correlations (p-value > 10^{-3}) were observed between the binding site properties and the performance of DiffDock-L, Vina pose sampling, or the differences between the two methods (Figure S9).

Protein similarity to DiffDock-L training data

Methods

New VS data curation process. The UniProt IDs corresponding to the protein PDB IDs in the DiffDock-L test set were obtained (Figure S10). The PubChem assay data (available by 10.02.2024) was acquired and preprocessed to standardize and clean up small molecules. Subsequently, only the assays with more than 32 ligands were retained, and the UniProt IDs of the tested proteins in the assays were obtained. The DiffDock-L test set and processed PubChem assays were matched using UniProt IDs. The resulting data were manually checked to ensure the quality of the protein structures and small molecules.

Docking. The protein structures of the new test set were downloaded from the Protein Data Bank (https://www.rcsb.org/), then loaded and prepared using the Protein Preparation Wizard in the Schrödinger Platform (version 2021-1). The preparation steps were conducted in a manner similar to those for DUDE-Z targets, including assigning bond orders, adding hydrogens, filling in missing side chains, removing water molecules, protonating residues at pH 7.4 using PROPKA, and minimizing hydrogens using the OPLS4 force field with restraints. The prepared protein structures served as input for both DiffDock-L and Vina. Docking with both programs employed the same scripts and settings as for DUDE-Z targets.

For reference, the prepared protein structures were loaded in the Schrödinger platform (version 2021-1) for docking with Glide version 9.0. The small molecules were prepared with LigPrep to assign ionized state at pH 7.4 and energy-minimized in implicit solvent using the OPLS4 force field. The binding sites were set to 30x30x30 $Å^3$ around the corresponding co-crystallized ligands. Thirty poses were generated for each compound.

Results

New VS test set. We identified three targets dissimilar to DiffDock-L training data (with ECOD annotations not included in the training), each with a sufficient number of active compounds (≥ 32) (see Table S3).

VS performance. The performance difference between DiffDock-L and Vina pose sampling on the new test set was marginal (Figure S11). Given the limited availability of data for analysis and the comparable performance of the methods, it remains challenging to draw definitive conclusions about the influence of protein similarity to the DiffDock-L training data and the relative performance of the methods.
